# Gut-microbiota-mediated host immune modulation: mechanisms, pathological dysbiosis, and therapeutic frontiers

**DOI:** 10.3389/fcimb.2026.1905445

**Published:** 2026-08-19

**Authors:** Pradeesh Babu, Vidhya Prakash, Suja Subhash, Muralidharan Vanuopadath, Jayalekshmi Haripriyan, Karthika Rajan, Avani Anu Geetha, Sandhya P., Geetha B. Kumar, Bipin G. Nair, Aravind Madhavan

**Affiliations:** School of Biotechnology, Amrita Vishwa Vidyapeetham, Kollam, Kerala, India

**Keywords:** dysbiosis, immune modulation, metabolites, microbiota, therapeutics

## Abstract

The mammalian immune system has evolved in constant dialogue with its diverse microbiota, forming an ecological and molecular partnership that is fundamental to health. This review examines how microbial communities shape immunity across developmental and functional axes, the immunological consequences of dysbiosis during infection and inflammatory disease, and emerging microbiota-targeted interventions. The host–microbiota–pathogen triad offers a framework to understand how commensals and pathogens compete for ecological niches and immune recognition, and how disturbances in this balance can cascade into chronic inflammation or infection. Microbial metabolites such as short-chain fatty acids, secondary bile acids, and tryptophan derivatives act as key bioactive intermediaries translating microbial activity into host immune architecture, influencing epigenetic programming, cellular differentiation, and mucosal barrier function. These interactions orchestrate tolerance toward commensals while maintaining effector readiness against pathogens, particularly through regulatory T cell (Treg)–Th17 balance, B cell education, and Immunoglobulin A (IgA) responses. When perturbed, as in infections caused by *Clostridioides difficile, Klebsiella pneumoniae, Salmonella enterica*, or *Listeria monocytogenes*, the ensuing dysbiosis reinforces immune dysfunction in a self-perpetuating cycle. Therapeutic frontiers now extend beyond conventional antimicrobial strategies to include live biotherapeutics, bacteriophage therapy, fecal microbiota transplantation, and metabolite-based (postbiotic) interventions. Future efforts must reconcile inter-individual microbiome variability with precision medicine, integrating metagenomic and metabolomic profiling to design safe, effective, and personalized microbiota-centered therapeutics.

## 1 Introduction: the dynamic interplay of host, microbiota, and immune system

The mammalian host is not a solitary entity but rather a complex ecosystem, co-inhabited by a vast and diverse consortium of microorganisms collectively termed the microbiota. The gut microbiota is predominantly composed of bacteria (10^14^ microbial cells), along with significant populations of archaea, viruses, primarily bacteriophages, and eukaryotic microorganisms such as fungi and protozoa (Sender et al., 2016; Wagner et al., 2020). Within the bacterial populations, the phyla comprising *Firmicutes, Bacteroidetes, Actinobacteria, Verrucomicrobia*, and *Proteobacteria* together account for the majority of microbial biomass (Yang et al., 2020). These microbial communities, predominantly residing in the gastrointestinal tract but also present on other mucosal surfaces and the skin, engage in a continuous and intricate dialogue with the host. This interaction is fundamental to numerous physiological processes, most notably the development, education, and function of the host immune system (Belkaid and Hand, 2014; Almansour et al., 2025). This review delves into the multifaceted mechanisms by which the human gut microbiota modulates host immune responses through microbial components and metabolites, immune signalling pathways, and host-microbe interactions as it is currently the best-characterized regulator of systemic immunity.

It explores the consequences of disruptions in this symbiotic relationship (dysbiosis) on inflammatory, metabolic, infectious, and other diseases (Huttenhower et al., 2012) and examines the burgeoning field of host microbiota targeted therapeutic strategies such as prebiotics, probiotics, postbiotics, engineered microbial therapeutics, fecal microbiota transplantation, and precision microbiome-based interventions that are aimed at enhancing host defense and restoring immune homeostasis (Nazir et al., 2025). The review uniquely emphasizes on the host-microbiota-pathogen triad and integrates recent advances in multi-omics, artificial intelligence -assisted microbiome analysis, and personalized microbiome medicine to provide a comprehensive understanding of microbiota-mediated immune regulation. The primary objective of the review is to collectively summarize on current knowledge in microbiome mediated immune responses, identifying existing research gaps, challenges, and analyse future opportunities for translating these findings into microbiome-targeted therapeutics.

The mammalian host and its microbiota have co-evolved over millions of years, establishing a profound mutualistic relationship that is indispensable for immune system development and function. This intricate dialogue ensures that the host immune system can differentiate between beneficial commensals and harmful pathogens, maintaining a delicate balance crucial for health. Specialized immune pathways, including the development of regulatory T cells and the production of immunosuppressive cytokines, have emerged to manage constant microbial exposure while retaining robust defenses against true threats (Derrien et al., 2023). Concurrently, microbes have developed strategies to modulate host immunity, ensuring their persistence and producing metabolites beneficial to the host.

Apart from its metabolic functions, commensal microorganisms residing in the gut contributes to host nutrition by synthesizing essential micronutrients such as vitamin K and B complex vitamins including cobalamin, riboflavin and folate that are absorbed by the host and required for a balanced nutritional health (LeBlanc et al., 2013), though the extent to which microbiota-derived vitamins contribute to host vitamin status remains incompletely understood.The development of nutritional homeostasis is required for promoting the growth of commensal organisms and preventing invasion by pathogenic species. Regulating the immune system development and function involves early microbial colonization resulting in maturation of both innate and adaptive immune responses and enhancing immune tolerance towards commensal organisms (Belkaid and Hand, 2014). Colonization by the commensal microbes confers resistance to the growth of pathogenic species by competing for nutrients and epithelial binding sites and stimulating host defense mechanisms such as antimicrobial peptide and IgA production (Pickard et al., 2017).

Disruptions to this finely tuned host-microbiota relationship, termed dysbiosis, can have far-reaching consequences, contributing to a range of immune-mediated diseases and increased susceptibility to infections (Fan and Pedersen, 2021). Factors such as antibiotic use, dietary changes, and stress can alter microbial composition, compromising gut barrier integrity, dysregulating immune responses, and reducing colonization resistance against pathogens (Koh et al., 2016; Duyvejonck et al., 2021). Understanding these complex interactions within the host-microbiota-pathogen triad is paramount for developing effective therapeutic strategies aimed at restoring immune homeostasis and enhancing host defense against bacterial infections and other diseases (Buffie and Pamer, 2013).

While this review highlights the role of intestinal microbiota as the best-characterized regulator of systemic immunity, other site-specific microbiota also contributes to immune homeostasis and disease. The respiratory, oral, skin, and urogenital microbiota are among the few that engage in local crosstalk with epithelial and immune cells, influencing barrier integrity, tolerance to commensals, and susceptibility to infection or inflammatory pathology. Although a comprehensive analysis of these ecosystems is beyond the scope of this article, we briefly highlight key parallels and distinctions where relevant to emphasize that microbiota–immune interactions are a general principle of host biology rather than a phenomenon restricted to the gut.

## 2 The host-microbiota-pathogen triad: a foundation for health and disease

The health status of an organism is profoundly influenced by the dynamic interplay within what can be conceptualized as the Host-Microbiota-Pathogen Triad (Thiennimitr et al., 2011). This framework underscores the continuous and reciprocal interactions among three key players: the host, with its epithelial barriers and sophisticated immune system; its resident commensal microbiota, a diverse assemblage of bacteria, viruses, fungi, and archaea that are generally beneficial or neutral; and potential pathogens, which are microbes capable of causing disease (Baümler and Sperandio, 2016). The gut flora, often referred to as a “forgotten organ, “ plays a critical role in this triad (O’Hara and Shanahan, 2006). In a healthy state, the host and microbiota maintain a balanced relationship, with the microbiota aiding in immune system development and providing colonization resistance against pathogens thereby contributing to immune tolerance (Ullah et al., 2024). However, this balance can be disrupted by factors like antibiotics, diet changes, or stress, leading to dysbiosis (Munita and Arias, 2016; Pandit et al., 2025). Dysbiosis compromises the gut barrier, dysregulates immune responses, and reduces colonization resistance, creating opportunities for pathogens to cause diseases such as inflammatory bowel disease, metabolic syndrome, allergies, and increased susceptibility to infections (Ezenabor et al., 2024; Shen et al., 2025). This often becomes a vicious cycle where infection worsens dysbiosis, therefore, therapeutic interventions should consider the entire host-microbiota-pathogen triad, focusing on restoring a healthy microbiota in addition to direct antimicrobial or anti-inflammatory treatments (Nazir et al., 2025).

### 2.1 Evolutionary co-development: mutualism and immune tolerance

The intricate and largely harmonious relationship observed between hosts and their resident microbiota is not a recent phenomenon but the product of millions of years of mutualistic co-development. This shared evolutionary journey has exerted profound selective pressures on both the host and its microbial symbionts, leading to sophisticated immune mechanisms capable of managing constant and extensive microbial exposure at mucosal surfaces. A key evolutionary imperative was the development of immune tolerance towards the vast numbers of harmless or beneficial commensals, while simultaneously retaining a robust capacity to defend against true pathogens. This evolutionary pressure drove the emergence and refinement of specialized regulatory immune pathways, including the development of regulatory T cells, tolerogenic dendritic cells (DCs), and the production of immunosuppressive cytokines like interleukin-10 (IL-10) and transforming growth factor-beta (TGF-β) (Belkaid and Hand, 2014), the sentinels of the innate immune system, evolved to discern subtle molecular differences between commensal-derived Microbe-Associated Molecular Patterns (MAMPs) and pathogen-derived PAMPs, allowing for nuanced responses (Allegretti et al., 2021). Furthermore, physical barriers (e.g., mucus layers, epithelial tight junctions) and chemical defenses (e.g., antimicrobial peptides (AMPs), secretory IgA (sIgA) at mucosal surfaces were optimized to limit microbial translocation and maintain compartmentalization (Brown et al., 2018).

Concurrently, commensal microbes evolved sophisticated strategies to adhere to host tissues, efficiently utilize host-derived nutrients, actively modulate or evade host immune responses to ensure their persistence, and produce metabolites that are often beneficial to the host (Ivanov and Littman, 2010; Belkaid and Hand, 2014). The symbiotic host-commensal relationships that develop, particularly after birth, are fundamental for enabling normal metabolic processes, comprehensive immune development, and effective anti-infectious defenses, all contributing to overall gut homeostasis and mucosal integrity (Tanaka and Nakayama, 2017; Zheng et al., 2020).

The stark reality of this co-dependence is vividly illustrated by studies involving germ-free animals. These animals, raised in a sterile environment devoid of any microbial contact, exhibit severe underdevelopment of their immune structures (such as Gut-Associated Lymphoid Tissues, GALT) and profound functional deficiencies in their immune responses (Rhee et al., 2004; Round et al., 2011). This underscores the indispensable role of the microbiota in educating and shaping the host immune system. The immune system’s remarkable ability to differentiate between commensal MAMPs and pathogenic PAMPs is a direct outcome of this co-evolutionary fine-tuning. This discrimination is not merely based on the presence or absence of a particular MAMP but likely involves a complex integration of contextual cues, the concentration of the microbial signal, and the presence of associated danger or safety signals from the microenvironment. Co-evolution would have strongly selected for hosts capable of tolerating beneficial microbes while vigorously eliminating harmful ones, based on these nuanced interpretations. The concept of the ‘hygiene hypothesis, ‘ which posits that reduced exposure to a diverse range of environmental and commensal microbes in early life may contribute to the rising incidence of allergic and autoimmune diseases in industrialized nations, can be partly understood through the lens of this disrupted co-evolved immune education (Okada et al., 2010; Stiemsma et al., 2015).

## 3 The microbiota: an indispensable architect of immune system development

The commensal microbiota actively and indispensably instructs and modulates the development, maturation, and lifelong function of both the innate and adaptive branches of the host immune system (Guo et al., 2016; Ullah et al., 2024). The human gut microbiota, in particular, is increasingly recognized as a potential controller of overall wellness and disease states (Afzaal et al., 2022). In the realm of innate immunity, the constant, low-level stimulation provided by commensal microbes serves to prime innate immune cells, including macrophages, neutrophils, and DCs, keeping them in a state of physiological alertness, ready to mount a rapid and effective response upon encountering a pathogen (Abt and Artis, 2013). Microbes directly contribute to the fortification of epithelial barriers by inducing epithelial cells to produce protective mucus, a variety of AMPs (such as defensins and RegIIIγ), and by reinforcing the tight junctions that seal the paracellular space between epithelial cells (Gudej et al., 2021). Furthermore, the microbiota critically influences the maturation, recruitment, and functional polarization of innate immune cells, for example, by promoting the development of anti-inflammatory macrophage phenotypes and by modulating the activation of inflammasomes, multi-protein complexes crucial for sensing pathogens and cellular stress (Chen et al., 2023; Yilmaz, 2024).

With respect to adaptive immunity, the microbiota is essential for the proper development of GALT, the primary site of immune induction in the gut (Mörbe et al., 2021). It is a principal driver of T cell differentiation. Specific groups of bacteria, such as certain *Clostridia* species or *Bacteroides fragilis*, are known to induce the differentiation of Tregs, largely through the production of SCFAs like butyrate, which are vital for establishing and maintaining immune tolerance (Ivanov et al., 2009; Arpaia et al., 2013). Conversely, other bacteria, like segmented filamentous bacteria (SFB), are potent stimulators of effector T cells, such as T helper 17 (Th17) cells, which play a critical role in mucosal barrier defense against specific types of pathogens (Ivanov and Littman, 2010).

SFBs are commensal Gram-positive, spore -forming bacteria that attach to the epithelial cells of the small intestine without causing disease (Ivanov et al., 2009). SFB have become a popular model for studying the crosstalk between the microbiota and the immune system, specifically for understanding the regulation of mucosal immunity and intestinal homeostasis by the commensal microbes. The microbiota also promote the maturation of intestinal immune system and governs B cell development and is a major stimulus for the production of sIgA, an antibody that coats luminal microbes, preventing their adherence to the epithelium and facilitating their clearance (Canales-Herrerias and Cerutti, 2023). Additionally, microbial signals shape the T cell receptor repertoire and influence the mucosal homing patterns of lymphocytes (Belkaid and Hand, 2014).

In essence, the microbiota continuously educates the immune system, contributing to gut homeostasis through a complex interplay of metabolic, immune, and anti-infectious processes (Zheng et al., 2020). It acts as a persistent, “live-in” adjuvant, constantly fine-tuning immune readiness and responsiveness in a manner distinct from the acute, often damaging, responses triggered by pathogenic encounters. This ongoing, low-level stimulation is beneficial, maintaining a state of “controlled inflammation” necessary for rapid pathogen response and barrier integrity (Hooper et al., 2012; Belkaid and Hand, 2014). Consequently, early-life microbial colonization represents a critical window for this immune education. Disruptions during this formative period, such as those caused by Caesarean section delivery, formula feeding instead of breastfeeding, or early and frequent antibiotic use, can have long-lasting consequences on immune development and may predispose individuals to a range of immune-mediated diseases later in life (Bokulich et al., 2016; Yasmin et al., 2017; Lathakumari et al., 2024).

### 3.1 Microbiota-driven modulation of innate immunity

The innate immune system, the body’s first line of defense, is profoundly shaped and regulated by the resident microbiota. This constant interaction ensures a state of preparedness against pathogens while maintaining tolerance to commensal organisms. Microbial signals, ranging from structural MAMPs to secreted metabolites, program the function of key innate immune cells, fine-tune critical signaling pathways, and can even induce long-lasting epigenetic changes (Belkaid and Hand, 2014; Belkaid and Harrison, 2017).

#### 3.1.1 Neutrophil priming and functional modulation

Commensal microbes play a dual role in regulating neutrophil activity. On one hand, low-level, continuous exposure to MAMPs such as Lipopolysaccharides (LPS) or peptidoglycan primes neutrophils through Toll-like receptor (TLR) and NOD-like receptor (NLR) activation (Vinolo et al., 2011). This priming enhances their recruitment efficiency to sites of infection and augments their phagocytic capacity, ensuring a swift and effective response during encounters with pathogens (Maslowski et al., 2009; Fachi et al., 2019).

On the other hand, microbial metabolites exert crucial suppressive control to prevent excessive neutrophil activity and collateral tissue damage (Deshmukh et al., 2014). Butyrate, an SCFA predominantly produced by commensal bacteria like *Clostridia* and *Roseburia*, functions as an HDAC (histone deacetylases) inhibitor. In neutrophils, this leads to reduced overproduction of reactive oxygen species(ROS), diminished NETosis (the formation of neutrophil extracellular traps), and suppressed secretion of pro-inflammatory cytokines like IL-8 and TNF-α (Cunha et al., 2017). This restraint is vital for limiting immunopathology (Villena et al., 2011). Conversely, acetate, another SCFA produced by bacteria such as *Bifidobacterium* and *Prevotella*, can enhance the expression of antimicrobial genes in neutrophils, partly through histone acetylation and signaling via the G protein-coupled receptor GPR43 (Yasuda et al., 2024). Specific commensals, like certain *Lactobacillus* species, can further modulate neutrophil function through receptor-specific pathways, for instance, by enhancing Dectin-1-mediated phagocytosis of fungal pathogens like *Candida* (Kennedy et al., 2007). This programming highlights a sophisticated “double-edged sword” mechanism: the microbiota primes neutrophils for enhanced pathogen response but simultaneously employs metabolites to actively suppress their potentially damaging activities, thus preventing immunopathology during homeostasis. Dysbiosis leading to a deficiency in butyrate-producing bacteria might result in neutrophils that are either insufficiently primed for defense or are excessively inflammatory, thereby contributing to chronic inflammatory conditions or impaired infection control (Villena et al., 2011).

#### 3.1.2 Macrophage polarization (M1/M2) driven by microbial signals

Microbial signals are critical determinants of macrophage phenotype and functional polarization, broadly categorized into pro-inflammatory (M1-like) and anti-inflammatory/tissue-reparative (M2-like) states. The microbiota fine-tunes this polarization through specific receptor-ligand interactions and metabolic reprogramming.

SCFAs, particularly butyrate and propionate (produced by commensals such as *Clostridia* and *Bacteroides*) actively promote an anti-inflammatory M2-like state in macrophages. They achieve this by activating G protein-coupled receptors like GPR109A and GPR43, and by inhibiting HDACs (Wang et al., 2023). These actions lead to increased production of anti-inflammatory cytokines IL-10 and TGF-β, and enhanced expression of arginase-1 (ARG1), an enzyme associated with M2 macrophage (Liu et al., 2018). Polysaccharide A (PSA) from *B. fragilis* further reinforces this M2 polarization via TLR2-dependent IL-10 induction (Coradduzza et al., 2023; Kawai et al., 2024).

While promoting anti-inflammatory responses, the microbiota also ensures macrophages can mount effective pro-inflammatory responses when necessary. However, it also provides regulatory mechanisms, for example, secondary bile acids, produced by the metabolic action of *Clostridium* species on host primary bile acids, can suppress the activation of the NLRP3 inflammasome (a key driver of IL-1β production) through activation of the TGR5 receptor. Simultaneously, SCFAs can boost the phagocytic efficiency of macrophages against pathogens like *Salmonella* and *Escherichia coli*. This demonstrates the microbiota’s capacity to not just induce a generic “anti-inflammatory” or “pro-inflammatory” state but to precisely tune macrophage functional polarization. The balance of M1/M2 macrophages is critical in diverse pathological contexts, including cancer, atherosclerosis, and wound healing (Chen et al., 2024). Therefore, the composition of the gut microbiota and its metabolic output represent key determinants of this balance, profoundly influencing disease progression and resolution (Belkaid and Hand, 2014; Guo et al., 2016; Ma et al., 2025).

#### 3.1.3 Microbial influence on type I interferon and inflammasome pathways

Commensal microbes are crucial for fine-tuning the activity of cytosolic immune sensors, including those involved in Type I interferon (IFN-I) production and inflammasome activation, thereby preventing aberrant or excessive inflammation.

*Bacteroides thetaiotaomicron*, a prominent gut commensal, has been shown to suppress IFN-I responses. It can achieve this through SCFA-mediated inhibition of HDACs involved in the STING (Stimulator of Interferon Genes) pathway, a key pathway for IFN-I production in response to cytosolic DNA. Additionally, *B. thetaiotaomicron* may compete for cytosolic DNA sensors, further dampening IFN-I signaling (Platt et al., 2021).

Inflammasome modulation by the microbiota is highly metabolite-specific and can be bidirectional. While certain pathobionts, like specific strains of *E. coli*, can activate inflammasomes such as NLRC4 or NLRP3 via their flagellin or LPS respectively, commensal-derived products often exert inhibitory effects. For instance, butyrate produced by commensal *Clostridia* can inhibit NLRP3 inflammasome assembly and activation, partly by blocking the activity of HDAC11, which is involved in regulating NLRP3 expression (Jiang et al., 2020). Furthermore, secondary bile acids can inhibit NLRP3 inflammasome activation via TGR5 signaling (Guo et al., 2016; Liu et al., 2024).

The microbiota’s regulation of IFN-I and inflammasome pathways represents a critical control point for antiviral immunity and responses to cellular damage or certain bacterial pathogens, respectively (Levy et al., 2015; Swanson et al., 2019). Dysregulation in these pathways, potentially stemming from dysbiosis, can lead to autoinflammatory conditions, autoimmune diseases, or impaired clearance of viral infections (Gopalakrishnan et al., 2018a). Consequently, therapeutic strategies that target Stimulator of interferon genes (STING) or inflammasome pathways, which are being explored for cancer immunotherapy or autoinflammatory diseases, might have their efficacy significantly influenced by an individual patient’s gut microbiota composition and its metabolic activity (Tacconelli et al., 2018; Wang et al., 2018; Kang et al., 2024).

#### 3.1.4 Dendritic cell maturation and antigen presentation: a microbial influence

Dendritic cells are crucial antigen-presenting cells that initiate and shape adaptive immune responses, playing a key role in differentiating between immunity and tolerance. Commensal microbes significantly influence DC function, generally programming them to promote immune tolerance towards the resident microbiota and dietary antigens. SCFAs, particularly propionate and butyrate, foster the development of tolerogenic DCs in the gut, characterized by CD103 expression, which produce retinoic acid (RA) and TGF-β—essential factors for differentiating naïve T cells into Foxp3+ Tregs (Ullah et al., 2024). Similarly, PSA from *B. fragilis* activates TLR2 on DCs, leading to the production of the anti-inflammatory cytokine IL-10, further contributing to a tolerogenic environment (Chaudhari et al., 2025). Lamina propria DCs, constantly exposed to microbial products, maintain a state of relative hypo responsiveness to commensal antigens through mechanisms like butyrate-mediated HDAC inhibition and the up-regulation of negative regulators such as Interleukin-1 Receptor-Associated Kinase M (IRAK-M), while still retaining the ability to respond robustly to pathogens (Zhao and Elson, 2018). These microbiota-educated DCs, after sampling antigens in the lamina propria, migrate to draining mesenteric lymph nodes where they present these antigens to T cells under tolerogenic conditions (e.g., in the presence of IL-10, TGF-β, and RA, and with low co-stimulation), thereby enforcing systemic immune balance and preventing unwanted reactions to harmless gut contents (Kang et al., 2024). Such microbiota-educated DCs are pivotal orchestrators of peripheral tolerance, and their capacity to induce Tregs and maintain hypo-responsiveness to commensal antigens is vital for preventing autoimmune and inflammatory reactions in the gut, with defects in these interactions increasingly implicated in the pathogenesis of inflammatory bowel disease (IBD) and cancer (Tan et al., 2016; Çİn et al., 2024; Kang et al., 2024; Wang et al., 2025).

#### 3.1.5 Regulation of natural killer cell activation and cytotoxicity

Natural Killer (NK) cells are innate lymphocytes critical for early defense against viral infections and tumor cells. The activity of NK cells is also subject to regulation by the commensal microbiota.

Microbiota-derived signals can stimulate IL-15 production by both DCs and intestinal epithelial cells (IECs) (Ganal et al., 2012). IL-15 is a key cytokine for NK cell development, maturation, survival, and priming. This stimulation enhances NK cell IFN-γ secretion and overall readiness, preparing them for rapid responses to viral threats. Specific microbial metabolites further fine-tune NK cell effector functions. For example, acetate, a SCFA, has been shown to amplify NK cell cytotoxicity, at least in part via GPR41-dependent up-regulation of granzyme B. Conversely, certain tryptophan metabolites, produced by commensals like *Lactobacillus* species, can activate the Aryl Hydrocarbon Receptor (AhR) on NK cells. AhR activation in this context tends to suppress excessive NK cell activity, which may be important for preventing autoimmunity or immunopathology driven by over-active NK cells (Zelante et al., 2013; Yoo et al., 2020). This dual regulatory influence—priming for enhanced function while also providing mechanisms for restraint—demonstrates that the microbiota fine-tunes NK cell activity to achieve a balance between effective immunosurveillance against infected or transformed cells and the prevention of excessive inflammation or autoimmune reactions. The efficacy of NK cell-based immunotherapies, which are being developed for cancer treatment, might therefore be significantly influenced by the patient’s gut microbiota composition and the profile of its metabolic products, such as acetate and tryptophan catabolites (Pérez Marc et al., 2025).

#### 3.1.6 Key microbial metabolites and their innate immunomodulatory roles

Microbial metabolites are a primary currency of communication between the microbiota and the host immune system (Postler and Ghosh, 2017). These structurally diverse small molecules, derived from microbial fermentation of dietary components or host-produced substances, can directly interact with host cells and signaling pathways, thereby translating the composition and activity of the microbial community into specific host immune responses.

##### 3.1.6.1 Short-chain fatty acids: acetate, propionate, and butyrate

SCFAs, predominantly acetate, propionate, and butyrate, are crucial microbial metabolites produced from the bacterial fermentation of dietary fibers in the colon. Acetate, the most abundant SCFA, provides energy to peripheral tissues, enhances reactive oxygen species production in neutrophils via GPR43 signaling, promotes antimicrobial gene expression through histone acetylation, and contributes to NK cell cytotoxicity. Propionate plays a role in gluconeogenesis and acts as an immunomodulator by promoting T reg induction through HDAC inhibition and GPR43 signaling, while also reducing TLR4 signaling in dendritic cells, leading to a less inflammatory state (Canales-Herrerias and Cerutti, 2023). Butyrate is a primary energy source for colonocytes and a potent HDAC inhibitor, which increases IL-10 and decreases IL-12 production in macrophages, promotes M2 macrophage polarization via GPR109A, and reduces NLRP3 inflammasome activation. Furthermore, butyrate is essential for maintaining epithelial barrier integrity by promoting tight junction protein expression, stabilizing hypoxia-inducible factor (HIF), and modulating IL-18 release (Baiou et al., 2021). A recent study has shown that the acetate derived from microbial metabolism can induce CD8+ T cell response against Influenza A virus infection (Tacconelli et al., 2018). Collectively, SCFAs help maintain a low luminal pH, inhibiting pathogen growth, and promoting mucus synthesis by goblet cells (Gaudier et al., 2004; Tan et al., 2014).

##### 3.1.6.2 Secondary bile acids

Primary bile acids synthesized by the host liver are metabolized by gut bacteria into secondary bile acids, such as deoxycholic acid (DCA) and lithocholic acid (LCA). These molecules have significant immunomodulatory roles. They can signal through nuclear receptors like farnesoid X receptor (FXR) and membrane receptors like TGR5 on immune and epithelial cells (Ridlon et al., 2014). Activation of TGR5, for instance, can lead to decreased NLRP3 inflammasome activity and increased IL-10 production by macrophages (Guo et al., 2016; Zhao et al., 2018). Secondary bile acids also play a crucial role in colonization resistance against certain pathogens, notably *Clostridioides difficile*, by inhibiting its germination and growth (Ivanov et al., 2009; Rahman et al., 2024). Secondary bile acid derivatives such as 3-oxoLCA (3-oxolithocholic acid) and isoalloLCA (isoallolithocholic acid) produced by gut microbiota plays a major role in regulating intestinal immune homeostasis. 3-oxoLCA directly inhibits the transcription factor RORγt, and suppresses the differentiation of pro-inflammatory Th17 cells, whereas isoalloLCA acts through mitochondrial reactive oxygen species-dependent mechanisms in promoting the differentiation and function of Treg cells (Hang et al., 2019). By reducing Th17 responses and enhancing Treg activity, these metabolites help sustain the balance between inflammation and immune tolerance. This has identified microbial bile acid metabolites as therapeutic targets for immune-mediated disease therapy.

##### 3.1.6.3 Tryptophan catabolites

Dietary tryptophan can be metabolized along two distinct routes. Gut bacteria (e.g., *Lactobacillus* spp., *Clostridium* spp.) convert tryptophan directly into a variety of bioactive indole derivatives (e.g., indole-3-aldehyde, indole-3-propionic acid), many of these compounds are ligands for the AhR. Concurrently, kynurenine and downstream kynurenine-pathway metabolites are generated predominantly by the host via indoleamine 2, 3-dioxygenase (IDO1) and tryptophan 2, 3-dioxygenase (TDO); although some gut bacteria can modulate these host-derived metabolites, the microbiota is not considered a major direct source of kynurenines. Activation of AhR in immune cells (ILCs, T cells, DCs) and epithelial cells can promote the production of IL-22 (crucial for barrier defense and tissue repair), decrease TNF-α production in macrophages, and enhance Treg cell function (Hezaveh et al., 2022).

##### 3.1.6.4 Polyamines

Polyamines such as spermidine, putrescine, and spermine are produced by both host cells and gut microbiota. They are involved in numerous cellular processes, including cell growth and proliferation. Immunologically, spermidine can enhance autophagy and decrease inflammasome activation. Some polyamines may also exert anti-inflammatory effects through HDAC inhibition and can modulate DNA methylation patterns by influencing the availability of S-adenosylmethionine (SAM), a universal methyl donor (Pirini et al., 2025; Soda, 2022).

This array of metabolites acts as a crucial chemical communication layer. The composition and metabolic activity of the microbiota, which are themselves heavily influenced by factors like diet and host genetics, are translated into specific host immune responses via these molecular messengers. They effectively form the “language” of host-microbe interaction. This understanding has fueled the development of “postbiotics”—the administration of these beneficial microbial metabolites or inactivated microbial components directly (Fiorucci and Distrutti, 2015). Postbiotics offer a potentially more direct and controllable means of immune modulation compared to live probiotics, as they can bypass challenges related to probiotic engraftment, viability, and inter-individual variability in metabolite production from a given probiotic strain (Salminen et al., 2021; Yilmaz, 2024). **Table 1** discusses about key metabolites in regulating T cell immunity.

**Table 1:** Key microbial metabolites regulating T cell subsets and immune responses.

Microbial metabolite	Source (microbiota)	T cell subset affected	Effect on polarization	Key references
Short-Chain Fatty Acids (SCFAs) (e.g., Butyrate, Propionate, Acetate)	*Clostridia*, *Bacteroides*	Tregs	Promotes differentiation and expansion of Foxp3^+^ regulatory T cells	(Arpaia et al., 2013; Furusawa et al., 2013)
Indole derivatives (e.g., Indole-3-aldehyde)	*Lactobacillus reuteri*, others	Th17, Tregs, Th22	AHR ligand; supports IL-22 production (Th22), balances Th17/Treg fate	(Zelante et al., 2013)
Polysaccharide A (PSA)	*B. fragilis*	Tregs	Induces IL-10-producing regulatory T cells, promoting anti-inflammatory responses	(Round et al., 2011)
Secondary Bile Acids (e.g., isoallo LCA)	*Clostridium* spp.	Tregs	Enhances Treg differentiation via mitochondrial reactive oxygen species	(Hang et al., 2019)
Desaminotyrosine (DAT)	*Clostridium* *orbiscindens*	Th1	Enhances type I IFN signaling and promotes Th1 responses against viral infection	(Steed et al., 2017)
Histamine	*Lactobacillus reuteri*	Th1, Th2	Suppresses Th1, promotes Th2 responses via histamine receptor signaling	(Gao et al., 2015)
Tryptophan metabolites (e.g., kynurenine)	Various commensals	Tregs, Th17	AHR ligands modulate T cell responses, potentially enhancing Tregs and limiting Th17	(Cervantes-Barragan et al., 2017)

The table summarizes the major microbial metabolites that help in regulating immune responses, specifically subsets of T-cell immunity.

#### 3.1.7 Epigenetic reprogramming of innate immune cells by microbial metabolites

Beyond acute signaling, microbial metabolites can induce durable, long-lasting changes in immune cell function through epigenetic modifications. These modifications alter gene expression patterns without changing the underlying DNA sequence, effectively “programming” immune cells for altered responsiveness.

- Histone Deacetylase Inhibition: As previously mentioned, SCFAs like butyrate and propionate are potent HDAC inhibitors. By inhibiting HDACs, they increase the acetylation of histone proteins (e.g., H3 and H4) associated with DNA. This acetylation generally leads to a more open chromatin structure, enhancing the accessibility of gene promoters to transcription factors. This mechanism is crucial for upregulating genes involved in immune regulation, such as the Foxp3 gene (master regulator of Tregs, where butyrate-mediated acetylation of conserved non-coding sequences stabilizes Treg lineage commitment) and the IL-10 gene (amplifying anti-inflammatory signals in macrophages and dendritic cells). The epigenetic specificity of these metabolites is noteworthy; for example, butyrate may preferentially target HDAC3 in macrophages but HDAC1 in T cells, leading to cell-type-selective functional outcomes (Zimmerman et al., 2012; Joensen et al., 2015).
- Aryl Hydrocarbon Receptor (AhR) Activation: Tryptophan catabolites (e.g., indole-3-aldehyde, kynurenine) produced by the microbiota serve as ligands for the aryl hydrocarbon receptor (AhR). Upon binding, AhR translocate to the nucleus, dimerizes with ARNT (AhR nuclear translocator), and binds to xenobiotic response elements (XREs) in the promoter regions of target genes. This drives the transcription of genes like IL-22, essential for epithelial barrier defense and repair, and various detoxification enzymes such as CYP1A1 (Chen et al., 2024).
- Polyamine and Folate Metabolism: Polyamines, such as spermidine (which can be derived from bacteria like *Bifidobacterium*), can further modulate the epigenetic landscape. Spermidine can inhibit histone acetyltransferases (HATs) under certain conditions and induce autophagy-related genes by promoting the dephosphorylation of the transcription factor TFEB. Moreover, polyamines and microbial folate metabolism are interconnected with one-carbon metabolism, which influences the availability of S-adenosylmethionine (SAM). SAM is the universal methyl donor for DNA methyltransferases (DNMTs) and histone methyltransferases. Thus, microbial influences on SAM availability can alter DNA methylation patterns and histone methylation, both of which are critical epigenetic markers (Mentch et al., 2015; Soda, 2022; Woo and Alenghat, 2022; Cao et al., 2024).

Metabolite-driven chromatin modifications can establish long-term “trained immunity” or innate immune memory. This refers to a non-heritable adaptive state where innate immune cells, upon primary stimulation (e.g., by microbial products), become hyper-responsive or hypo-responsive to secondary, unrelated stimuli (Saeed et al., 2014). This epigenetic programming by the microbiota suggests that early-life microbial exposures or sustained dietary patterns that shape the microbiota can have enduring effects on an individual’s immune responsiveness and susceptibility to inflammatory diseases throughout their life (Rondeau et al., 2020; Jing et al., 2025). This provides a strong mechanistic basis for how diet and microbiota can exert long-term influences on health. Although, evidence for trained immunity is demonstrated in both murine and human studies; however, the molecular mechanisms have been significantly characterized in murine models.

### 3.2 Microbiota’s guiding hand in adaptive immunity

The human microbiota, especially the gut ecosystem, significantly influences the adaptive immune system. Rather than being passive residents, commensal bacteria actively guide the development, differentiation, and function of immune cells. It is well established that the gut microbiota modulates multiple cellular signaling pathways that sustain host homeostasis (Ivanov and Honda, 2012; Kim et al., 2025). This modulation is crucial for lowering the risk of chronic inflammatory diseases such as arthritis, cardiovascular disease, and inflammatory bowel disease. Recent research indicates that signals from the microbiota can direct T cell polarization, B cell education, and the formation of immunologic memory (Ivanov et al., 2009; Zhao et al., 2017; Pellon et al., 2025). The entire immune system, including both humoral and cellular immunity, benefits from commensals because they are essential for immune training and functional calibration (Belkaid and Hand, 2014). These interactions are vital for maintaining immunological tolerance, mucosal protection, and adjusting the host response to infections, vaccines, and immunotherapies (Pellon et al., 2025). Among the key elements, secretory immunoglobulin A (sIgAs) in the adaptive immune system plays a major role in epithelial defense and is directly involved in regulating microbiota composition. This section discusses the diverse effects of microbiota on adaptive immunity, focusing on T-cell fate determination, mucosal IgA production, and the maintenance of long-term immune surveillance.

#### 3.2.1 Balancing T cell fates: Treg/Th17 differentiation and broader Th cell polarization

The gut microbiota profoundly influences the differentiation of CD4+ T cells into various functional subsets, including Tregs and pro-inflammatory Th1 and Th17 cells. Commensal bacteria act as crucial immunological instructors, orchestrating both pro-inflammatory responses and immune tolerance, thereby ensuring immunological homeostasis. Central to this balance are CD4+ Foxp3+ Tregs, which significantly impact the maturation and differentiation of the adaptive immune system. Naïve CD4+ T cells are highly responsive to microbial cues, differentiating into either effector or regulatory T cells based on the specific microbial antigens and metabolites they encounter. This interaction also underpins the development of immunological memory, where antigen-experienced T cells provide a more rapid and robust response upon subsequent exposure. However, imbalances in the gut microbiota, known as dysbiosis, can disrupt this delicate equilibrium, leading to the activation of T cells and the initiation of various immune disorders (David et al., 2014; Gopalakrishnan et al., 2018a; Ling et al., 2024).

Specific microbial drivers play distinct roles in T cell polarization. *Clostridium* species (clusters IV and XIVa) are key in promoting Treg differentiation by enhancing TGF-β signaling and producing butyrate, which upregulates Foxp3 expression (Atarashi et al., 2013). Similarly, *B. fragilis* induces IL-10+ Tregs through its capsular polysaccharide A (PSA), which activates TLR2 on dendritic cells. Conversely, other microbes drive pro-inflammatory T helper cell responses; for instance, Segmented Filamentous Bacteria (SFB) induce Th17 cell polarization by stimulating dendritic cells to produce IL-6 and IL-23, while *Prevotella copri* is associated with Th17 cell expansion and systemic inflammation (Ivanov et al., 2009; Belkaid and Harrison, 2017). Beyond direct microbial action, T helper polarization is also modulated by various metabolites, including tryptophan catabolites, secondary bile acids, and branched-chain fatty acids, which interact with host receptors to bias T cell differentiation. Examples include *Lactobacillus reuteri*, which metabolizes tryptophan into AhR ligands that promote IL-22-producing cells, and beneficial bacteria like *Akkermansia muciniphila* and *Faecalibacterium prausnitzii*, which induce Tregs and suppress pro-inflammatory Th1 responses through SCFA production and mucin degradation (Atarashi et al., 2013; Zelante et al., 2013; Derrien et al., 2023). This intricate interplay, where specific microbial taxa and their metabolites promote either immune tolerance or activation, underscores the complexity of T cell fate decisions within the gut and highlights the potential for microbiota-targeted immunotherapies in managing inflammatory bowel disease, autoimmunity, and cancer. Emerging data further emphasize strain-specific effects, demonstrating that even within the same genus, different bacterial strains can elicit contrasting immune responses depending on their metabolite profiles, localization, and host genetics, reinforcing the microbiota’s critical role as a regulator of immune fate decisions within the gut-associated lymphoid tissue (GALT). **Table 1** provides a list of major microbial metabolites that help in regulating T-cell based immune responses.

#### 3.2.2 Educating B cells and driving mucosal IgA production

The gut bacteria play pivotal roles in B-cell development, activation, differentiation and function via T cell-dependent or independent pathways (Heidari et al., 2024). These pathways are essential for immunological tolerance, barrier integrity, and the mutualistic host-microbiota relationship. These activities can be mediated by bacterial components or metabolites in the gut, through direct and indirect effects.

The largest pool of immunoglobulin in the body is found at mucosal sites, and within the gut in particular, where SIgA plays a crucial role as an anti-infective barrier and supporting maintenance of a healthy microbiota (Routy et al., 2023). A major fraction of mucosal IgA plasma cells develops from B cells in the mucosal-associated lymphoid tissue (MALT) upon activation (Lycke and Bemark, 2017). B-cell maturation and education for IgA production depends on a series of well-organized events including class switch recombination (CSR) via T-cell-dependent or independent pathways, affinity maturation, and terminal differentiation to plasma cells (Canales-Herrerias and Cerutti, 2023; Routy et al., 2023).

Most B cell education for IgA production mainly occurs in organised mucosal lymphoid tissues like Peyer’s patches, mesenteric lymph nodes, and isolated lymphoid follicles. These areas are essential for germinal center (GC) reactions, which facilitate clonal expansion, affinity maturation, and diversification of IgA producing B cells, especially in response to various antigens from pathogens and commensals. Peyer’ s patches continually sample gut lumen microbes such a*s B. fragilis, E. coli, and Lactobacillus species* (Gao et al., 2024). B cell class switching from IgM to IgA needs signals from both T- Dependent (TD) and T- Independent (TI) pathways, with strong help from helper T cells particularly T follicular helper (Tfh) cells and dendritic cells (Cerutti, 2008). Cytokines like TGF- β, IL- 21, and BAFF/APRIL (B-cell activating factor/A proliferation inducing ligand), secreted by these cells, activate the IgA gene locus and promote class switch recombination (CSR) (Xu et al., 2006; Liu et al., 2023). Tfh cells assist in TD induction through interactions like CD 40- CD 40 L and cytokine release, especially TGF- β and IL- 21. They also help in affinity maturation, selecting high- affinity IgA clones, as shown with bacterial and non- bacterial oral antigens (Carreto-Binaghi et al., 2024). TI pathways, often activated by microbial signals, depend on dendritic cell- derived BAFF and APRIL to drive IgA CSR in the absence of T cell support (Fleming et al., 2022). Polysaccharide antigens from bacteria such as *Salmonella* spp., *Streptococcus pneumoniae*, and *Neisseria meningitidis*, along with microbial ligands like LPS and CpG DNA, stimulate B cells via pattern recognition and B cell receptors, promoting T- independent IgA CSR (Chaudhari et al., 2025). These bacterial signals trigger B cells to undergo CSR to IgA by engaging Toll- like receptors and activating dendritic cells, which secrete factors like BAFF and APRIL, ensuring strong mucosal immunity even without T cell help. Additionally, dendritic and epithelial cells produce factors such as retinoic acid and TSLP (Thymic stromal lymphopoietin) that help shape the local environment for IgA induction (Tezuka and Ohteki, 2019).

#### 3.2.3 Establishing long-term vigilance: microbiota and immunological memory

Microbiota are essential for immune vigilance, shaping both innate and adaptive memory from birth by educating the immune system through pattern-recognition receptor activation and the imprinting of memory phenotypes. This leads to “trained immunity” in monocytes, macrophages, and innate lymphoid cells, where microbial products like β-glucan and peptidoglycan induce epigenetic remodeling and heightened responsiveness to subsequent infections, lasting for months (Afzaal et al., 2022). Commensal-derived antigens and metabolites, such as SFB (Segmented filamentous bacteria) capsular antigens, *B. fragilis* polysaccharide A, and SCFAs like butyrate, promote the differentiation of long-lived effector and regulatory immune cells, facilitating antigen presentation and histone acetylation crucial for generating specialized memory T and B cells, including tissue-resident memory T cells (Trm) in the gut. SCFAs also support memory T cell survival and anti-inflammatory cytokine production, while the microbiota influences IgA+ memory B cell diversity, providing mucosal protection. Microbial molecules like polysaccharide A from *B. fragilis* engage TLR2 on Treg cells, promoting mucosal tolerance and long-term immune regulation, generating systemic cues that orchestrate peripheral immune homeostasis and enhance vigilance against pathogens (Round et al., 2011; O’Toole et al., 2017). By combining immunological memory, B-cell education, and T-cell balance, the microbiota acts as a master regulator of adaptive immunity, shaping the Th1/Th2/Th22 spectrum, sustaining tolerance and pathogen resistance, and fine-tuning T cell development through microbial antigens and metabolites that promote class switching, B-cell activation, and secretory IgA production, creating a protective mucosal barrier. These signals support durable immune vigilance through tissue-resident memory T cells, IgA+ memory B cells, and trained innate populations, highlighting the microbiota as an active architect of immune homeostasis and memory, underscoring the therapeutic potential of diet, probiotics, prebiotics, and microbiome-targeted interventions to enhance vaccine efficacy, prevent chronic inflammation, and improve outcomes in infection and cancer immunotherapy.

In a clinical trial in melanoma patients receiving anti–PD-1 checkpoint blockade, higher relative abundance of *A. muciniphila* and *Bifidobacterium* spp. has been associated with improved clinical response (Gopalakrishnan et al., 2018b; Routy et al., 2018), while in mouse models, oral *Bifidobacterium* supplementation and *B. fragilis* colonization has each been shown to enhance anti-PD-L1 and anti-CTLA-4 efficacy, respectively (Sivan et al., 2015; Vétizou et al., 2015). Fecal microbiota transplantation from immunotherapy responders has also been shown to restore anti–PD-1 responsiveness in previously refractory melanoma patients in early-phase clinical trials (Davar et al., 2021).

**Figure 1:**
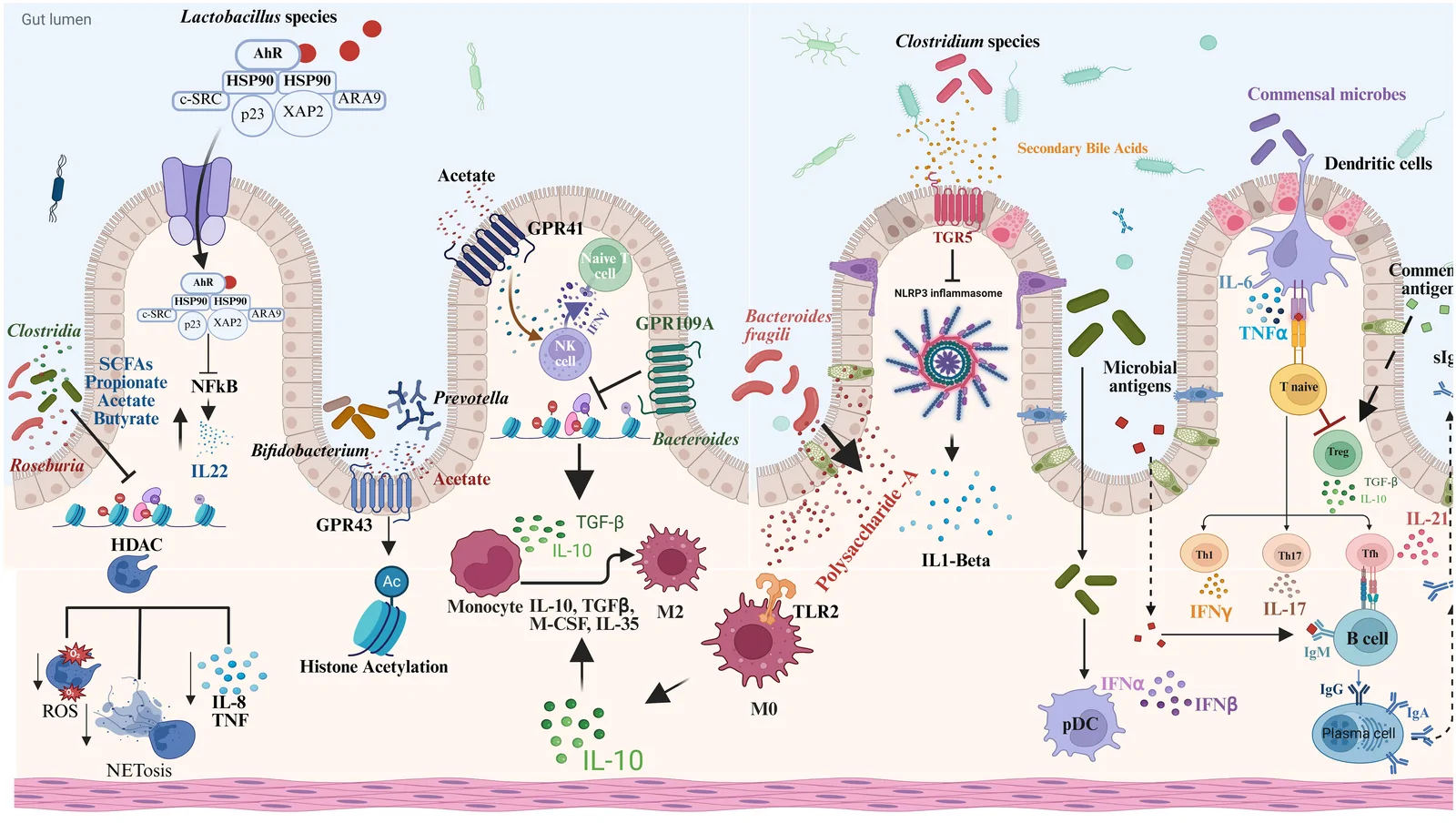
Microbiota-mediated immune modulation: key metabolites and pathways.

**Figure 1** provides a detailed illustration of major microbiota-mediated immune modulation, key metabolites, and their associated pathways.

## 4 Molecular dialogues: mechanisms of microbiota-host immune crosstalk

### 4.1 Gut-associated lymphoid tissue: the primary interface for immune education

The largest mass of lymphoid tissues in the body is Gut-Associated Lymphoid Tissue, accounting for approximately 70% of all immune cells. This is very crucial for creating a balanced immune status. Human gut-associated lymphoid tissue is composed of multi-follicular Peyer’s patches located in the ileum, the vermiform appendix, and numerous isolated lymphoid follicles (ILFs) distributed throughout the length of the intestine. GALT plays a key role in maintaining immune balance by helping the body tolerate harmless substances like food proteins and beneficial gut microbes, while simultaneously defending against harmful pathogens. This balance prevents chronic inflammation and supports overall health through a continuous process of immune regulation and education (Jørgensen et al., 2021) as an immune interface. Its architecture consists of two main components: organized lymphoid tissues, like Peyer’s patches, which initiate immune responses, and diffuse lymphoid tissues spread throughout the intestine, which contain mature immune cells, each playing a distinct role in educating the immune system.

Organized GALT are lymphoid aggregates that serve as the primary sites for antigen exposure and the initiation of immune responses. These includes Peyer’s patches (PPs) located in the small intestine, mesenteric lymph nodes (MLNs), and isolated lymphoid follicles (ILFs) found throughout the intestine. The primary function is to process antigens and instruct lymphocytes on how to respond, effectively. Peyer’s patches (PPs) are unique feature of GALT, acting as the primary link for antigen surveillance. PPs are covered by a specialized single layer of cells called the Follicle-Associated Epithelium (FAE) which are characterized by the presence of Microfold (M) cells (Jung et al., 2010).

These cells are distinct from regular epithelial cells as they actively endocytose and transport particulate antigens from the gut lumen to the immune cells in the underlying lymphoid tissue. This function also makes M cells a vulnerable point in the mucosal barrier where pathogen can use for their entry. This is defended by the subepithelial dome (SED) present just beneath the FAE, consisting of dendritic cells and macrophages (Wagner et al., 2020).

This multi-layer defense strategy of GALT offers surveillance and critical vigilance in the gut (Jung et al., 2010). Diffuse GALT includes scattered immune cells, mainly mature lymphocytes, located within the intestinal epithelium and the underlying lamina propria (LP) where neutralization of pathogens and regulation of inflammation occurs.The migration of activated lymphocytes from the inductive sites to the effector sites is the mechanism of mucosal (Depommier et al., 2019).

### 4.2 Commensal bacteria actively shaping host responsiveness via TLR modulation

Commensal bacteria play a crucial role in actively shaping host immune responses through the modulation of Toll-like receptor (TLR) signaling pathways. TLRs recognize microbial molecules, function as key sensors at mucosal surfaces, allowing the host to distinguish between harmful pathogens and beneficial commensals (Rakoff-Nahoum et al., 2004). Interaction of TLRs with commensal bacteria typically results in controlled signaling which maintains immune tolerance and tissue homeostasis, distinct from the strong pro-inflammatory responses triggered by pathogens. This balance is achieved through mechanisms such as TLR desensitization or tolerance, involving downregulation of receptor expression and induction of negative regulators. These prevents excessive inflammation in spite of the consistent presence of microbial ligands in mucosal environments (Qiu et al., 2024). The influence of commensal bacteria with TLR signaling in epithelial and immune cells strengthen barrier integrity by promoting tight junction formation, secretion of antimicrobial peptides, and mucus production, which limits microbial invasion. Additionally, specific commensal-derived molecules, such as polysaccharide A from *B. fragilis*, interact with TLR2 to drive regulatory T cell responses that further strengthen immune tolerance and prevent tissue-damaging inflammation (Ramakrishna et al., 2019). Structural variation and difference in affinity of microbial ligands enable TLRs to discriminate between commensals and pathogens, thus modulating downstream signaling pathways, including NF-κB and MAPKs, that can promote symbiotic relationship. TLRs play a critical role in the intestine by balancing immune tolerance to commensal microbes with the initiation of inflammatory responses against pathogens, thereby maintaining mucosal homeostasis and preventing excessive inflammation. Alterations in Toll-like receptor signaling or changes in the composition of the microbiota can disrupt immune balance, compromise barrier function, and increase vulnerability to disease, underscoring the critical importance of interactions between commensal microbes and TLR pathways (Qiu et al., 2024). The understanding of TLR signaling mechanism aids in novel treatment strategies that modulate these pathways for a healthy and better management of inflammatory diseases.

### 4.3 Orchestrating homeostasis: microbiota’s role in the tolerance-inflammation equilibrium

The human gastrointestinal tract serves as a primary site for interaction between microorganisms and the host immune system, where the gut microbiota supports host physiology by aiding in nutrient degradation, fermentation, and pathogen defense through competition and secretion of antimicrobial peptides (Takiishi et al., 2017). A balanced immune environment in the gut depends on signals from microbiota that regulate the equilibrium between proinflammatory IL-17-producing Th17 cells and immunosuppressive Foxp3+ regulatory T cells, with gut microbial metabolites such as SCFAs—including acetate, butyrate, and propionate—playing key regulatory roles by acting as ligands for G protein-coupled receptors and inhibitors of histone deacetylases to promote anti-inflammatory, tolerogenic immune responses essential for maintaining intestinal immune homeostasis (Ivanov and Honda, 2012; Rooks and Garrett, 2016). Butyrate and propionate, key SCFAs produced by gut microbiota, directly inhibit prolyl hydroxylase enzymes, leading to stabilization of hypoxia-inducible factor (HIF)-1α in intestinal epithelial cells, which is crucial for maintaining gut homeostasis (Hang et al., 2019; Wang et al., 2021). Stabilized HIF regulates transcription of target genes responsible for barrier integrity—including mucin genes (MUC2, MUC3), tight junction proteins (e.g., claudin1), antimicrobial peptides (DEFB1), and factors involved in epithelial repair (ITF) (Wang et al., 2021; Singh et al., 2023). Under normoxic conditions, HIF-1α is degraded; but in hypoxia, HIF-1α accumulates, binds to HIF-1β, and activates protective gene expression (Hang et al., 2019). SCFA-induced HIF stabilization also promotes CD4+ T cell and innate lymphoid cell (ILC) production of IL-22, further supporting epithelial barrier function and mucosal healing, even under physiologic and inflammatory hypoxia (Yang et al., 2020). Thus, microbiota-derived SCFAs—primarily butyrate—modulate the host-microbe crosstalk by regulating HIF activity, which is central to barrier maintenance, immune tolerance, and protection against intestinal disease (Wang et al., 2021).

In addition to short-chain fatty acids, the gut microbiota produces other important immunomodulatory metabolites such as indole derivatives from tryptophan and polyamines from dietary arginine. Indole derivatives enhance goblet cell proliferation, antimicrobial peptide secretion, and mucin production, thereby strengthening the intestinal epithelial barrier (Zelante et al., 2013). Maintaining intestinal immune balance depends on the regulation of pro- and anti-inflammatory cytokines; for instance, IL-10 produced by regulatory T cells prevents excessive inflammation, while IL-17A from Th17 cells mediates immune defense but can also drive inflammation and tissue damage. Certain bacterial genera, like *Bacteroides*, cause a decline in IL-17A production, whereas others such as *Prevotella* increase IL-17A levels (Round and Mazmanian, 2009). Additionally, cytokines like IL-22 from Th17 and ILC3 cells contribute to defense against extracellular pathogens, and IL-12, modulated by *Lactobacillus* strains, drives Th1 responses critical for intracellular pathogen immunity (Kim et al., 2025). Thus, the gut microbiota and intestinal cytokines engage in a complex, dynamic interaction that shapes host immune responses and influences health and disease outcomes.

## 5 When balance is lost: dysbiosis in the context of bacterial infections

The human microbiota encompasses an exemplary symbiotic diversity of microorganisms, predominantly bacteria, fungi, archaea, viruses, and protozoa colonizing the gastrointestinal (GI) tract, with the leading microbial load followed by the oral, skin, respiratory, and genitourinary tracts. The colon harbors approximately 3.8 × 10¹³ bacterial cells, which is roughly proportionate to the number of human cells in an average 70 kg adult (Gupta et al., 2022a). The majority of the microbiota is represented by the phylum *Firmicutes*, including *Clostridium* spp. and *Bacillus* spp., while *Lactobacillus* and members of *Proteobacteria* such as *E.coli* and *Klebsiella* spp. are relatively less abundant (Kundu et al., 2017).

The interplay between the relative abundance of these taxa within the GI tract serves as a crucial determinant for maintaining eubiosis, signifying a balanced and healthy microbial ecosystem. It is well established that gut microbial colonization varies with age, mode of delivery, geographical location, dietary diversity, genetic background, sanitary conditions, and host immune tolerance (Yatsunenko et al., 2012).

However, dysbiosis—defined as the imbalance in microbial composition—is characterized by an overrepresentation of detrimental microbes that disrupt intestinal homeostasis, leading to infections and inflammatory disorders. Although gut dysbiosis is multifactorial, pathogen-specific dysbiosis has gained particular focus, highlighting how pathogenic taxa exploit host and microbial interactions to drive disease states.

### 5.1 Pathogen-specific dysbiosis and bidirectional interactions

Pathogen-specific dysbiosis represents a more severe perturbation, characterized by the overgrowth of a single pathogen or a pathogenic consortium that disturbs the gut microbial equilibrium (Bokulich et al., 2016). This phenomenon often involves either an excessive proliferation of opportunistic pathogens or the depletion of beneficial commensals. Pathogen-driven dysbiosis can manifest through several mechanisms:

a) Pathogen invasion into systemic circulation due to compromised epithelial barriers in the gut, skin, or respiratory tract; b) Chronic inflammatory activation by persistent pathogens that manipulate host immune responses, leading to disease progression; c) Metabolic disruption, wherein pathogens produce harmful metabolites that interfere with host metabolic pathways and inhibit the synthesis of SCFAs by commensals; and d) Enhanced virulence expression, allowing pathogens to outcompete beneficial microflora for ecological niches and nutritional resources (Stecher and Hardt, 2011).

The host–pathogen–microbiota axis is inherently bidirectional, wherein host factors such as immune status, diet, and genetic background influence microbial composition, while pathogens reciprocally alter host physiology through local or systemic infections that trigger inflammation, barrier dysfunction, and metabolic shifts (Pickard et al., 2017). Such interactions are exemplified by infections involving *C. difficile*, *Salmonella enterica*, *K. pneumoniae*, and *Listeria monocytogenes*, all of which can cause profound alterations in the gut microbiota and host–pathogen dynamics.

### 5.2 *Clostridioides difficile*: exploiting a disrupted ecosystem

*Clostridioides difficile* infection (CDI), a predominant global health threat manifested by hospital-associated diarrhea, represents one of the most prominent examples of pathogen-specific dysbiosis, marked by profound alterations in the gut microbiota and host–pathogen interactions (Gupta et al., 2022b). CDI pathogenesis is primarily characterized by a disrupted gut ecosystem, often emerging after antibiotic therapy. Antibiotic-induced dysbiosis can decimate SCFA-producing commensal bacteria, compromising gut barrier integrity. Several studies have shown that CDI impacts bile acid metabolism, resulting in accumulation of primary bile acids such as cholate and taurocholate, which promote *C. difficile* spore germination and vegetative growth, while secondary bile acids like deoxycholate, which inhibit vegetative cells, are reduced (Sorg and Sonenshein, 2008; Theriot et al., 2014).

The reduction in bacterial diversity also frees ecological niches and nutrients, supporting *C. difficile* proliferation (Chang et al., 2017). The pathogen exploits spore germination in response to bile acids and low oxygen, and secretes exotoxins (TcdA and TcdB), which damage enterocytes and induce inflammation and pseudo membrane formation (Voth and Ballard, 2005). Deoxycholate can enhance biofilm formation in *C. difficile*, and extracellular pyruvate from other commensals imported via transporters (CstA) may act as a key biofilm-promoting signal (Tremblay et al., 2021).

Persistent recurrence is further linked to host manipulation—stool samples from CDI patients showed elevated indole levels, which suppress beneficial flora intolerant to indole, thereby supporting *C. difficile* dominance (Darkoh et al., 2019). Moreover, co-culturing *C. difficile* with *Clostridium scindens* in bile acid media reduced pathogen abundance, even without bile acids, suggesting that metabolic reshaping of the gut environment can suppress *C. difficile* (Saenz and Villarreal, 2023).

Another mechanism of contact-dependent antagonism involves *B. thetaiotaomicron*, which inhibits *C. difficile* toxins via cell wall-associated glycans, preventing pathogen autolysis (Kitamoto et al., 2020). More comprehensive strategies beyond antibiotics and FMT have shown promise—for instance, butyrate supplementation proved most effective in controlling *C. difficile* compared to acetate or propionate in murine models (Huttenhower et al., 2012). Additionally, IL-22–driven host glycosylation facilitates *Phascolarctobacterium* species to outcompete *C. difficile* for nutrients, reinforcing colonization resistance.

These insights emphasize the intricate microbial–metabolic–immune crosstalk governing CDI and highlight the potential of targeted microbiome-based interventions.

### 5.3 *Salmonella enterica*: thriving in inflammation

*Salmonella*-Induced Inflammation and Commensal Disruption: *Salmonella* orchestrates host inflammation to the detriment of commensal microbiota, gaining a growth advantage. *S. enterica* serovars, such as *S. typhimurium*, induce robust inflammatory responses via Salmonella Pathogenicity Islands (SPIs), particularly SPI-1 and SPI-2, which encode type III secretion systems (T3SSs).SPI-specific effectors, including SopE and SipA, trigger pathogenesis through mechanisms such as actin pedestal formation and membrane ruffling, disrupting tight junctions and activating NF-κB, ultimately leading to polymorphonuclear cell (PMN) recruitment and pro-inflammatory cytokine release (Haraga et al., 2008; Stecher and Hardt, 2011). SPI-2 is crucial for intracellular survival and replication within macrophages and sustaining inflammation, especially during systemic infection, by activating ERK and STAT2 pathways and promoting a “cytokine storm” (Jennings et al., 2017).

*Salmonella* subverts the host’s inflammatory response to outcompete commensal microbiota. *S. enterica* utilizes host-derived ethanolamine and promotes the conversion of thiosulfate to tetrathionate, enabling anaerobic growth on ethanolamine, a nutrient inaccessible to most microbes (Thiennimitr et al., 2011). The hepatobiliary system’s unique microenvironment also contributes to chronic *Salmonella* persistence (Kurtz et al., 2020). *S. typhimurium*-induced mucosal inflammation increases luminal aspartate, and host-produced reactive oxygen species can lyse commensal bacteria, creating a favorable niche (Yoo et al., 2024).

Commensal iron metabolism, specifically by *B. thetaiotaomicron* through XusB, can modulate host-pathogen dynamics by acquiring enterobactin-chelated iron, which *Salmonella* can then re-acquire, evading host iron restriction (Baümler and Sperandio, 2016). Two small RNAs (sRNAs), SdsR and Spot 42, regulate *Salmonella* virulence by targeting hilD mRNA, promoting SPI-1 expression and invasiveness (Abdulla et al., 2023). Interestingly, mutations in global virulence regulators like barA/sirA, while diminishing virulence gene expression and weakening the inflammatory response, can paradoxically lead to extended shedding and long-term colonization during persistent infections.

Recent research indicates that *Salmonella Typhimurium* infection alters host lipid metabolism and immune responses via eicosanoid signaling and PPARα activation. Loss of PPARα or inhibition of fatty acid oxidation reduces inflammation and bacterial burden, highlighting the role of lipid-immune crosstalk in infection severity (Taddeo et al., 2024). Furthermore, our studies demonstrate that oxyresveratrol, a polyphenolic stilbene, enhances *Lactobacillus fermentum* survival and probiotic traits and, at sub-inhibitory levels, synergistically inhibits *Salmonella enterica* by targeting the virulence factor SrfJ, suggesting its potential as a gut-protective dietary supplement.

In summary, the literature consistently highlights the intricate molecular mechanisms by which *Salmonella* orchestrates and benefits from the host’s inflammatory response, often at the expense of the commensal microbiota. This understanding is crucial for developing novel therapeutic and preventive strategies against salmonellosis.

### 5.4 *Klebsiella pneumoniae*: colonization and infection in the dysbiotic gut

*Klebsiella pneumoniae is* an increasingly significant opportunistic pathogen, especially in the context of gut dysbiosis. The focus has shifted from *K. pneumoniae* merely being an opportunistic colonizer to an active participant in shaping the dysbiotic niche for its benefit. Studies have proved that infection with classical *K. pneumoniae* (cKp) induces gut dysbiosis displayed by reduced microbial diversity and depletion of key genera such as *Lachnospiraceae* and *Bifidobacterium* in strain-infected mice and altered the lung metabolome, particularly affecting caffeine-related pathways. These findings establish *Klebsiella*-driven modulation of the gut–lung axis, supporting the rationale for microbiota-targeted interventions in respiratory pathophysiology (Jiang et al., 2022). Another study in gut-lung axis also showed the impact of asymptomatic gut colonization by carbapenemase-producing *Enterobacterales* (CPE) in mice, inducing gut dysbiosis, notably by the depletion of *Muribaculaceae* and reduced SCFA, leading to fewer alveolar macrophages and conventional dendritic cells in the lungs, exacerbating the severity of *Pseudomonas aeruginosa* lung infections. FMT successfully reversed these immune and infection outcomes, highlighting the critical role of CPE-induced gut dysbiosis in modulating systemic immunity (Le Guern et al., 2023). Several reports also focused on the prevention of cKp propagation in the gut, where dietary carbohydrates (fibers) could critically regulate colonization and dissemination from the gut and aid in commensal bacterial recovery post-antibiotics, while simple carbohydrates, like lactulose, promoted the pathogen growth, highlighting dietary interventions as a potential preventative strategy against disseminated infections in vulnerable patients (Hecht et al., 2024). However, another observation claimed the pathogens’ ability to metabolize alternative carbon sources like mucin-derived fucose for colonizing the GI tract, overcoming host colonization resistance, competing with commensals like *E. coli* strain Nissle 1917, and to positively modulate virulence traits such as hypermucoviscosity and biofilm formation (Hudson et al., 2022). On the contrary, the dynamic state of capsule expression during gut colonization may critically modulate patient risk for subsequent *K. pneumoniae* infections. In a study, spontaneous capsule loss was observed during gut colonization in a pathotype-dependent manner, driven by insertion sequences or mutations within capsule operon genes. Such capsular variants exhibit significant fitness defects in a murine pneumonia model, indicating a direct impact on virulence (Unverdorben et al., 2025). It was noteworthy that chronic alcohol consumption induces significant gut microbiota and metabolome dysbiosis, including altered bacterial and fungal diversity and reduced secondary bile acids, which promoted *K. pneumoniae* intestinal colonization in mice, specifically, secondary bile acids like lithocholic acid being identified as a key mechanism facilitating *K. pneumoniae* proliferation and adhesion (Qiu et al., 2024). Several studies have also shown that the synergistic interaction with pathogens promotes *Klebsiella* virulence. This was evidenced by *Candida albicans* co-inhabitation, which could significantly exacerbate *K. pneumoniae*-induced colitis in mucositis mice, increasing mortality, gut leakiness, systemic inflammation, and liver pathology, facilitating systemic hyper-inflammatory responses by promoting gut translocation of microbial products (Panpetch et al., 2022). However, recent reports also suggest that cohabitation of *Klebsiella oxytoca* promoted *K. pneumoniae* decolonization in ampicillin-treated mice by degrading the antibiotic and facilitating commensal outgrowth. However, a high-fat/high-sugar western-style diet significantly impairs this pathogen clearance, indicating the impact of the aforementioned practice, impeding functional microbiota recovery and compromise colonization resistance against opportunistic pathogens (Almási et al., 2025). These insights underscore the critical interplay between diet, antibiotics, and interbacterial competition in shaping gut colonization outcomes.

### 5.5 *Listeria monocytogenes*: navigating host defenses and microbiota barriers

*Listeria monocytogenes*, a ubiquitous, intracellular pathogen causing listeriosis, employs multifaceted strategies for navigating the human digestive system, including evading chemical and physical barriers like stomach acid and bile, and utilizing bacteriocins (Oliveira et al., 2025). Earlier studies have shown the profound impact of the intestinal microbiota on host gene expression during *Listeria monocytogenes* infection, specifically focusing on microRNA (miRNA) and protein-coding gene regulation. A microbiota-dependent decrease in the expression of five specific miRNAs (miR-143, miR-148a, miR-200b, miR-200c, miR-378) upon *Listeria* infection, was analyzed through bioinformatic analysis, suggesting a broader miRNA-mRNA regulatory network, work thus influencing host transcriptional reprogramming in response to a human pathogen (Rolhion and Cossart, 2017). Studies have also shown that *L. monocytogenes* employs multiple, redundant mechanisms for dissemination from intestinal tissue to mesenteric lymph nodes (MLN) (Cossart and Lebreton, 2014). It was shown that Ly6C^hi^ monocytes are not essential for MLN colonization, and increasing circulating dendritic cells significantly enhanced bacterial burden in draining lymph nodes and spleen. Furthermore, both free-floating and cell-associated *L. monocytogenes* were observed within lymphatic vessels (Nowacki et al., 2025). Reports have also shown the effect of Lmo2776, a *Listeria* bacteriocin, which surprisingly limited intestinal colonization by targeting and reducing the abundance of the gut commensal *Prevotella copri*. However, deletion of Lmo2776 leads to a thinner mucus layer in mice and increased Listerial loads in a microbiota-dependent manner. Thus, phenocopied by *P. copri* pre-colonization, the pathogen selectively utilizes a commensal species to mitigate excessive inflammation, ultimately enhancing its infection. Several studies have demonstrated the contributions of autophagic pathways to inhibiting Listerial growth. While noncanonical autophagy does not significantly restrict bacterial proliferation, xenophagy, dependent on FIP200 and TBK1, restricts the growth of *Listeria* mutants, while wild-type *Listeria* actively evades this xenophagic capture in the host cytosol via its phospholipases (PlcA, PlcB) and ActA (Mitchell et al., 2015). *L. monocytogenes* is also reported to primarily evade host autophagy through the combined action of its virulence factors PlcA and ActA. While listeriolysin O (LLO) enables cytosolic access, mechanistically, PlcA was shown to directly block LC3 lipidation, needed for autophagosome biogenesis, revealing a novel bacterial strategy to subvert host immunity efficiently (Mitchell et al., 2015). Thus, Listeria’s modulation of mitochondrial and autophagic regulators suggests a broader strategy leveraging host metabolism. Flavin acquisition strategies of the riboflavin auxotroph *L. monocytogenes*, essential for its virulence, were also studied, where the pathogen necessitates *de novo* riboflavin synthesis for extracellular proliferation, by scavenging pre-formed FMN and FAD via the RibU transporter for intracellular replication within host cells. Ablation of *ribU* abrogates virulence, whereas disrupting FMN/FAD synthesis converted *L. monocytogenes* into an obligate intracellular pathogen (Rivera-Lugo et al., 2022). Recent studies have also elucidated internalin B (InlB) as the primary ligand for *Listeria* adhesion protein (LAP), a secreted acetaldehyde alcohol dehydrogenase (AdhE) crucial for *L. monocytogenes* intestinal and brain invasion. The interaction transformed the housekeeping enzyme AdhE into a surface-anchored pathogenic factor, thereby facilitating *L. monocytogenes* transmucosal trafficking (Rivera-Lugo et al., 2022). Additionally, *L.monocytogenes*’s invasin, InlB, also demonstrated its critical role in accelerating phagosomal escape and subsequent intracellular proliferation by subverting class III PI3K/Vps34 signaling. This promoted the rapid conversion of the *Listeria*-containing vacuole into an escape-conducive Rab7 late phagosome, highlighting a sophisticated manipulation of endocytic trafficking by the pathogen in the study (Cain et al., 2023). Overall, these insights refine our understanding of *L. monocytogenes* pathogenesis and underscore the need for therapeutic interventions in preventing listeriosis.

**Figure 2:**
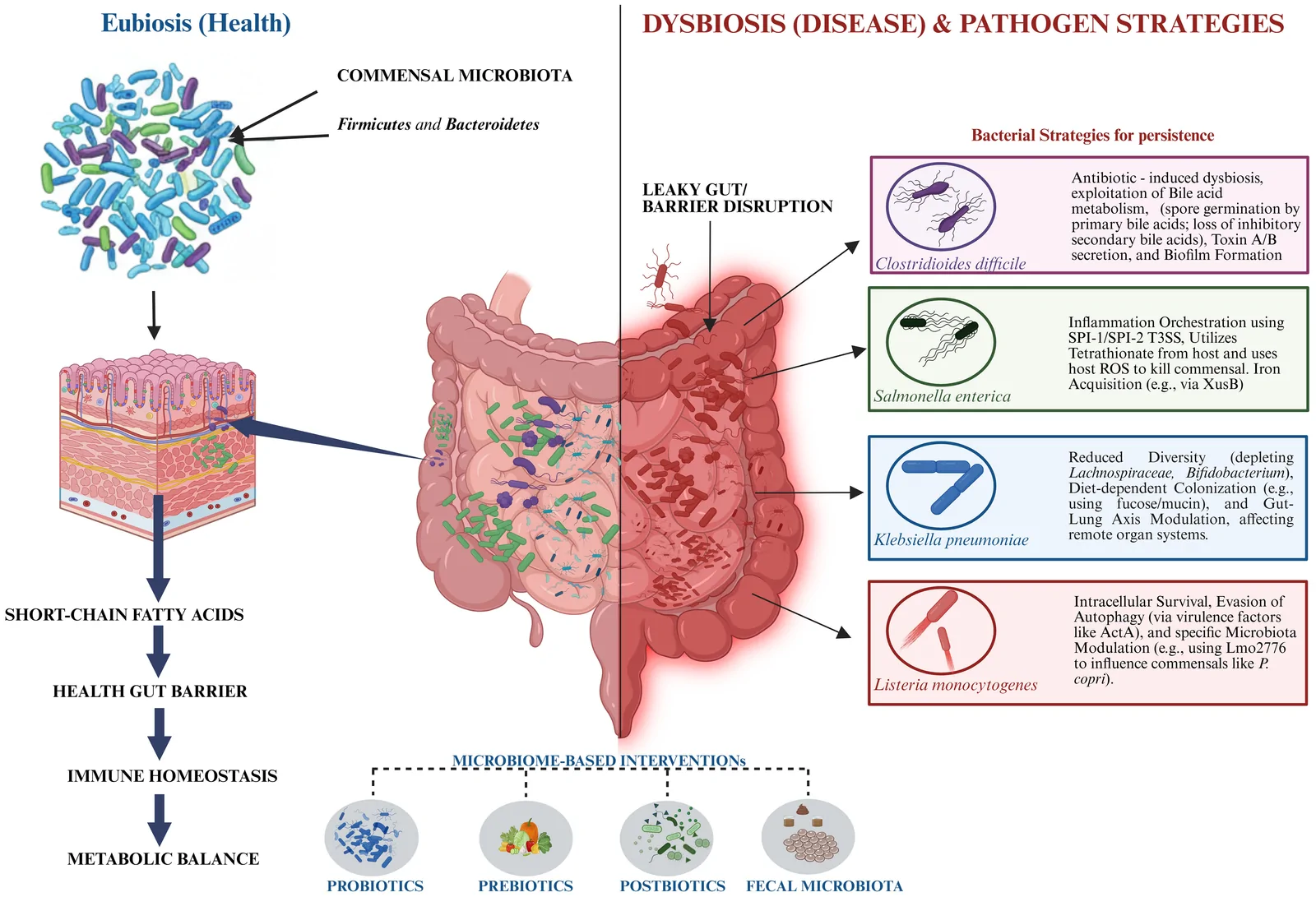
The host-microbiota-pathogen triad and dysbiosis cycle. A schematic overview depicting the communication between host, commensal microbiota and pathogen and how it is modulated in dysbiosis and the strategies adopted by pathogens for persistence.

Host-Microbiota-Pathogen Triad and the Dysbiosis Cycle as illustrated in **Figure 2**, explains the complex relationships between a host, its microbiota, and pathogens. It shows how a disruption in this balance leads to dysbiosis, perpetuating a cycle of imbalance and disease, and details factors influencing the triad and the mechanisms leading to detrimental relationships.

## 6 Live biotherapeutics: probiotics, engineered strains, and defined consortia

The term probiotics indicates live microorganisms that provide health benefits when taken in sufficient quantities, as proposed by the Food and Agriculture Organization/World Health Organization in 2002 (Suez et al., 2019). Their complex interactions are vital to maintain the intestinal homeostasis. Reports have indicated that the number of bacterial cells in the adult human gut microbiota is at least equal to the entire number of somatic and germ cells (Sender et al., 2016). Moreover, the genomes of these microbiota collectively contain almost 500 times as many genes as the human genome (Li et al., 2014). Human health could possibly be greatly enhanced by addressing the gut microbiota, and metagenomics research conducted over the last 20 years has found a wide variety of bacteria that could be used to create next-generation probiotics (O’Toole et al., 2017).Though there are numerous studies that have checked the potential clinical applications of probiotics to address several disease conditions, only very few have been discovered to be safe for ingestion. Initial evidence indicates that they improve both human and mouse glucose metabolism (Depommier et al., 2019; Gilijamse et al., 2020). Even though the consumption of probiotics is gaining popularity and increasing, conflicting results are also obtained in some of the clinical trials (Suez et al., 2019).

### 6.1 Nourishing beneficial microbes: prebiotics and synbiotics

Prebiotics are fiber-containing carbohydrates that the human intestine is unable to digest and solubilize. Prebiotics, however, are essential for improving host cell activities (Li et al., 2021). However, synbiotics are a combination of both probiotics and prebiotics. They synergistically support the growth and survival of good gut flora, which improves overall immunity and gastrointestinal health. Prebiotics have the potential to modify the immune response by changing the makeup of gut microbial communities or by producing microbial compounds such as SCFAs (Liu et al., 2022). Studies have shown that prescription of prebiotics together with lifestyle changes is ideal for the management of metabolic disorders and obesity (Ben Othman et al., 2023). In another study the prebiotic effect of inulin-type fructans in fecal microbiota was evaluated. The study showed that without altering the variety of fecal microbes, supplementing with inulin-type fructans produced higher quantities of fecal SCFA and had a notable bifidogenic impact (Birkeland et al., 2020). Similarly, reports have shown promising results in using synbiotics to treat Crohn’s disease, IBD, and ulcerative colitis (Martyniak et al., 2021).

### 6.2 Precision targeting: bacteriophage therapy

Despite the effectiveness, antibiotics kill gut flora randomly, which leads to dysbiosis and resistance problems (Awoniyi et al., 2023). Phage therapy has been recognized as an alternative to antibiotic treatments. Specially designed phage treatments that target pathogenic strains may reduce negative effects on good gut bacteria; however, there are still issues with phage transport and specificity (Zuo et al., 2018). Challenges like inadequate colonization and the potential for hazardous agent dissemination hamper traditional approaches like probiotics and fecal microbiota transplantation (Zuo et al., 2018; Karimi et al., 2024). The inability to precisely modify members of intricate microbial communities is one of the biggest drawbacks of the methods currently employed to modify the gut microbiota (Voorhees et al., 2020). However, two widely used approaches include engineered bacteria, or the ex vivo approach and phage therapy, *in-vivo* approach, which uses a concept of genetically modifying the microbiome to benefit from the rich microbial environment in the gut. Phages have been extensively investigated as a potential treatment for bacterial infections (Gordillo Altamirano and Barr, 2019; Kortright et al., 2019). However, they are now being used for the precision editing of gut microbiota. Unlike ‘ex vivo’ editing, the phage-based therapy will not alter the gut microbiota; instead, it directly edits the specific microbe, improving their half-life in the gut environment (Voorhees et al., 2020). Having been considered, phage-based treatments have the potential to be effective and promising methods of modifying the gut microbiota; nevertheless, further fundamental and preclinical research, along with well-planned randomized, double-blind, placebo-controlled trials, is needed to support the advancement of the area (Huttenhower et al., 2012; Hsu et al., 2019).

### 6.3 Ecosystem restoration: fecal microbiota transplantation—from rCDI to IBD, graft versus host disease, and cancer immunotherapy

Trillions of bacteria make up the intricate and comprehensive human gastrointestinal (GI) tract microbiota ecosystem. A disturbance of this delicate balance may result in several health problems, as it is vital to digestive health, preventing diseases, and overall wellness. In disorders where the gut microbiota is disturbed, fecal microbiota transplantation (FMT) has become a ground-breaking immunological strategy for regulating the host-microbe axis. This has been identified and is being used as an adjuvant therapeutic intervention for many diseases, especially with those conditions having dysbiosis as the primary reason (Heidari et al., 2024). FMT restores microbial biodiversity and balance by introducing fecal material from a healthy donor into the recipient’s alimentary tract (Wang et al., 2019). The application of FMT has been successfully proven in treating many bacterial infections, obesity, diabetes, autoimmune and inflammatory bowel disease (IBD), and many other disorders (Allegretti et al., 2021; Huang et al., 2022; Wu et al., 2023). Though many *in vitro*, *in vivo*, and clinical studies substantiate the potential of FMT in treating the above-indicated diseases, very limited studies are being done to prove their efficacy for GVHD and cancer immunotherapy (Routy et al., 2023; Reddi et al., 2025).

### 6.4 Metabolite-based strategies: the rise of postbiotics

Postbiotics are the byproducts of these microorganisms, in the form of metabolites, that exerts health benefits directly or indirectly in the host organism. As mentioned under 6.1 and 6.2, probiotics, prebiotics and synbiotics are connected directly or indirectly (in the form of health supplements) with the growth or survival of beneficial microorganisms in enhancing the gut function. Even though postbiotics have shown to enhance the gut microbiota, they are not considered as synbiotics (Klemashevich et al., 2014). Since they are not involved in the use of microorganisms directly or indirectly, they are safer than probiotics with minimal risks and shows same effects as probiotics. These metabolites are extracted through chemical or mechanical methods including solvent extraction, enzymatic extraction, heating and sonication. These metabolites are then extracted, isolated, dialyzed and characterized using different chromatographic techniques coupled with mass spectrometry strategies for their characterization (Singhal et al., 2015; Munita and Arias, 2016). Though probiotics, prebiotics and synbiotics showed promising results in clinical trials, the postbiotics formulations needs to be optimized for assessing their health benefits (Allegretti et al., 2021). The different class of postbiotics includes cell-free supernatants, exopolysaccharides, enzymes, cell wall fragments, bacterial lysates, metabolites produced by gut microbiota, and short-chain fatty acids. Reports have indicated that these molecules have shown potential immunomodulatory, antitumor, infection prevention, autophagy, wound healing, and antiatherosclerosis effects (Huttenhower et al., 2012).

Even though, postbiotics, probiotics, and FMT have shown significant therapeutic advantages, it poses certain limitations as evidenced from recent clinical studies. One such study reviewed by Sanders et al., observed the different human clinical trials of probiotics and concluded that certain bacterial strains are effective in treating particular disorders or conditions such as diarrhea associated with antibiotic administration and certain disorders of gastrointestinal tract. They have also indicated that the benefits of using probiotics cannot be generalized as clinical outcomes depend on the dose of administration, microbial strain, disease indication, and host-specific factors (Sanders et al., 2019). A similar study by the International Scientific Association of Probiotics and Prebiotics (ISAPP) proposed the definition of postbiotics and highlighted their potential advantages over probiotics, including improved safety, stability, and standardized production. Nevertheless, the authors observed that studies supporting postbiotics remains limited, with many studies restricted to preclinical models and in early-phase clinical trials, suggesting the need for large-scale randomized controlled studies (Salminen et al., 2021). FMT, also faces similar challenges, though FMT is highly effective for recurrent *Clostridioides difficile* infection, the high-end requirement for rigorous donor screening, standardized processing protocols, and long-term safety monitoring is a limitation (Cammarota et al., 2019). In a clinical trial conducted on patients with anti-PD-1-resistant metastatic melanoma, observed that FMT from immunotherapy-responsive donors, together with anti-PD-1 treatment, restored clinical responses in a subset of patients by modulating the gut microbiota and enhancing antitumor immune activity (Davar et al., 2021). Altogether, these studies indicate that therapies based on microbiota modulation have significant translational potential but require further investigation into the mechanistic aspect, optimization of clinical protocols, and large multicenter trials to optimize safety, efficacy, and patient selection.

**Figure 3:**
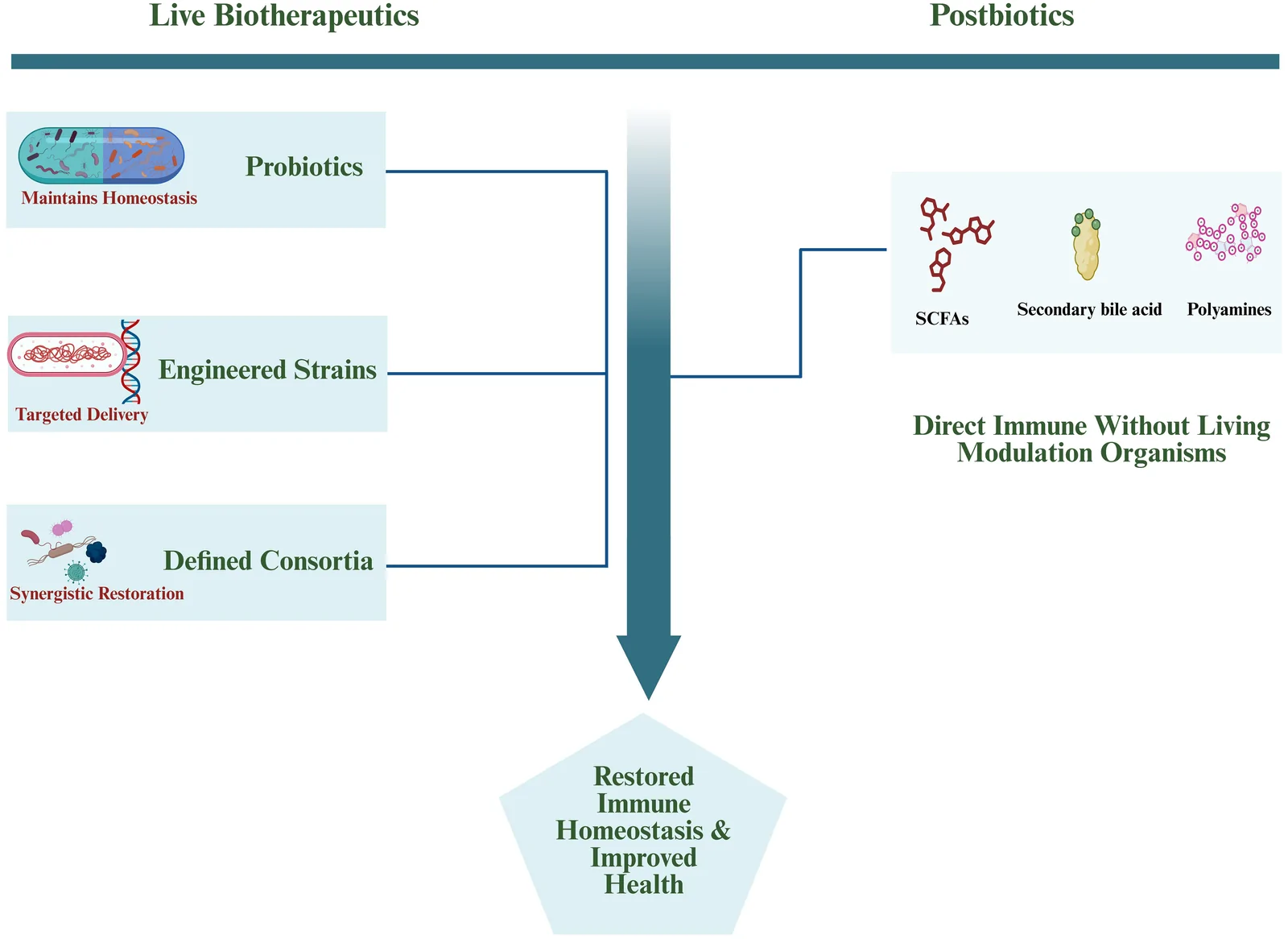
Therapeutic frontiers: modulating the microbiota to bolster host immunity. A schematic representation of how different types of microbiota modulation can lead to improved host immunity.

**Table 2** and **Figure 3** provide a comprehensive overview of microbiota-targeted adjunct therapies. Specifically, **Table 2** details these therapies, while **Figure 3**, “Therapeutic Frontiers: Modulating the Microbiota to Bolster Host Immunity, “ visually represents these therapeutic advancements.

**Table 2:** An overview of microbiota-targeted adjunct therapies.

SI. no	Type of therapy	Mode of action	Examples	Relevant clinical applications	References
1	Probiotics	Gut niche colonization, pathogen competition, immune modulation, barrier reinforcement, and host-microbe interactions	Species of -Lactobacillus, Bifidobacterium, Bacillus, Streptococcus, Enterococcus, Saccharomyces	Treating disorders- digestive, diabetes, inflammatory, cardiovascular, obesity, respiratory, disease affecting central nervous system	(Sarita et al., 2025)
2	Prebiotics	Selective stimulation of beneficial microbes through substrate supplementation that the microbes can utilize to stimulate host gut microbiota activation	Inulin, glucooligosaccharides, fructooligosaccharides, lactulose, derivatives of galactose and β-glucans	IBD, Crohn’s disease, Diabetes, Gastritis, depression, fibromyalgia, cystic fibrosis	(Birkeland et al., 2020; Jakubczyk et al., 2020; Gudej et al., 2021; Jackson et al., 2023; Çİn et al., 2024; Khademi et al., 2024; Khademi et al., 2024; Sarita et al., 2025)
3	Postbiotics	Immune modulation, infection prevention, lipid/cholesterol metabolism, antitumor effects, antioxidant activity	Cell free supernatants, Exopolysaccharides, Enzymes, Cell wall fragments, SCFA, Bacterial lysates and Metabolites	IBD, Ulcerative colitis, common infectious diseases, dermatitis	(Bodemer et al., 2017; Nocerino et al., 2017; Tomusiak-Plebanek et al., 2018)
4	Fecal Microbiota Transplantation	Transfer of healthy donor microbiota to restore microbial diversity.	fecal material from healthy human volunteers	Irritable bowel syndrome, Ulcerative colitis, Crohn’s disease, Autoimmune disorders, Multiple sclerosis	(Huang et al., 2022; Boicean et al., 2023; Porcari et al., 2023)

The table summarizes various microbiota targeted therapies for enhancing therapeutic efficacy.

## 7 Challenges, ethical considerations, and future directions

Despite the immense therapeutic promise of harnessing the microbiota, the translation of these innovative strategies from bench to bedside is fraught with significant challenges (Cani, 2018; Durack and Lynch, 2019; Proctor et al., 2019). These span biological complexity, regulatory hurdles, practical clinical integration, and the rigorous demands of evidence-based medicine (Thursby and Juge, 2017).

### 7.1 Navigating inter-individual microbiome variability: towards personalized medicine

A primary and profound challenge in the field is the inherent and extensive variability of the human microbiome. The notion of a single, universally “healthy” microbiome composition is a misconception; rather, healthy individuals exhibit a vast spectrum of microbial states that are compatible with health. This remarkable diversity is driven by a complex interplay of numerous factors, including:

- Host Genetics: An individual’s genetic makeup, particularly genes involved in immunity (e.g., Human Leukocyte Antigen (HLA) genes, genes encoding PRRs or cytokines) and metabolism, influences which microbes can successfully colonize and how the host responds to them (Bonder et al., 2016).
- Diet and Lifestyle: Long-term dietary patterns are a dominant force shaping the gut microbiota. Diets rich in diverse fibers tend to promote the growth of SCFA-producing bacteria like *F. prausnitzii*, which are often associated with gut health. Conversely, diets high in processed foods, animal fats, simple sugars, and low fiber, are typically associated with different microbial profiles that may be linked to inflammation and chronic disease (David et al., 2014). Lifestyle factors such as physical activity, stress levels, and hygiene practices also contribute to this process.
- Life History and Environmental Exposures: An individual’s history of antibiotic use, mode of birth (vaginal delivery versus Caesarean section, which influences initial microbial inoculum), infant feeding practices (breastfeeding versus formula), geographical location, and exposure to environmental microbes all have a lasting imprint on their microbial ecosystem (Yatsunenko et al., 2012).

This extreme inter-individual variability implies that a universal probiotic, live biotherapeutic product (LBP), or prebiotic intervention is unlikely to be effective for all individuals. A microbial strain that demonstrates therapeutic benefit in one group may fail to engraft—that is, establish stable, long-term colonization—in another because the recipient’s native microbiota outcompetes it for nutrients and ecological niches. Consequently, the future of microbiota-based therapeutics lies in personalized, data-driven approaches (Huttenhower et al., 2012). This vision entails leveraging advanced analytical tools such as metagenomic sequencing, to assess microbial composition and functional potential, and metabolomics, to quantify microbial metabolic output. These data can then be integrated using machine learning and artificial intelligence models to identify functional deficits or dysbiotic signatures within a patient’s microbiota, such as the overabundance of a pathobiont. Therapeutic strategies could then involve selecting or engineering a microbial strain—or a tailored prebiotic or postbiotic—designed to fill the identified ecological and functional void, thereby maximizing engraftment success, restoring function, and achieving clinical benefit (Page et al., 2015). The high inter-individual variability of the microbiota represents a major hurdle, necessitating a paradigm shift toward personalized interventions, underpinned by investments in bioinformatics, standardized methodologies for sample collection and analysis, and large-scale longitudinal studies to understand variability and build predictive models for individualized therapies.

### 7.2 Regulatory, safety, and manufacturing hurdles for live biotherapeutics

The use of living, replicating organisms as therapeutic agents introduces unique regulatory, safety, and manufacturing considerations that differ fundamentally from those for conventional static, small-molecule drugs or even traditional biologics like monoclonal antibodies (Morrow and Felcone, 2004).

#### 7.2.1 Safety concerns

- Risk in vulnerable populations: A foremost concern is the safety of LBPs in immunocompromised individuals, the elderly, or critically ill patients. A microbe that is innocuous in a healthy person could potentially cause opportunistic infections, bacteremia, or even sepsis in a host with a weakened or dysregulated immune system (Cordaillat-Simmons et al., 2020).
- Horizontal gene transfer (HGT): A significant long-term risk is the potential for transfer of genetic material (e.g., via plasmids or bacteriophages) from the administered therapeutic microbe to members of the recipient’s resident microbiota, or vice versa. This raises the alarming possibility of inadvertently spreading antibiotic resistance genes or virulence factors, especially if the LBP is engineered or if the chassis strain harbors such elements (Cordaillat-Simmons et al., 2020).
- Unintended immunological effects: Introducing a novel microbe, even a commensal, could trigger unwanted or unexpected inflammatory responses in some individuals. Conversely, it might induce long-term immune tolerance that could, in theory, impair the host’s ability to respond effectively to other infections or even to cancer cells (Cordaillat-Simmons et al., 2020).

#### 7.2.2 Regulatory and manufacturing hurdles

- Manufacturing complexity and consistency: Ensuring batch-to-batch consistency for a living product is exceptionally challenging. The viability, genetic stability, and functional potency of the microbes must be rigorously maintained throughout the manufacturing process and during storage. This often requires strict anaerobic (oxygen-free) manufacturing environments, specialized culture media, and sophisticated quality control measures, all of which add complexity and cost (Cordaillat-Simmons et al., 2020).
- Dosing and pharmacokinetics/pharmacodynamics (PK/PD): Unlike a traditional drug with predictable absorption, distribution, metabolism, and excretion (ADME) profiles, an LBP can replicate, colonize, and interact dynamically with the host environment. This makes its “dose” inherently dynamic and its PK/PD characteristics difficult to define using standard pharmacological models. Determining the appropriate initial dose, predicting its activity, persistence, and eventual clearance from the body are major challenges for regulators like the U.S. Food and Drug Administration (FDA) and the European Medicines Agency (EMA), which have had to develop new guidance documents specifically for LBPs (Cordaillat-Simmons et al., 2020).
- Characterization and Potency Assays: Defining and measuring the “potency” of a complex LBP, which may exert its effects through multiple mechanisms (e.g., metabolite production, immune modulation, competitive exclusion), is far more complex than for a single-molecule drug.

The “living” nature of LBPs, while offering unique therapeutic advantages such as self-replication, sustained local production of therapeutic molecules, and ecosystem modulation, simultaneously presents the most significant regulatory and manufacturing challenges (Rouanet et al., 2020). Close and continuous collaboration between researchers, industry stakeholders, and regulatory agencies is therefore essential to develop appropriate, scientifically sound, and adaptable frameworks for evaluating the safety, efficacy, and quality of LBPs, ensuring patient safety while fostering innovation in this rapidly evolving field.

### 7.3 Integrating microbiome profiling into clinical practice

The multi-omics datasets retrieved from metagenomics, metaproteomics, metatranscriptomics, and metabolomics studies can be integrated using sophisticated computational tools and AI, helps in understanding complex host-microbiome interactions. These machine learning algorithms can analyze huge, multidimensional datasets to identify microbial metabolites, their metabolic pathways, and immune responses elicited in the host. Such multidimensional analysis integrating different omics layers enable the discovery of potential biomarkers for disease diagnosis, prediction of disease progression and treatment response, and identification of novel therapeutic targets (Lloyd-Price et al., 2019; Proctor et al., 2019). In addition, AI-assisted prediction models can classify patients according to disease risk or therapeutic responses, based on individual microbiota profiles and thereby support the development of precision microbiome-based diagnostics and personalized therapeutic interventions (Schüssler-Fiorenza Rose et al., 2019).

For the vision of personalized microbiome-based medicine to become a reality, microbiome analysis must transition from being primarily a research tool to becoming a routine, accessible, and clinically validated diagnostic modality. Several practical barriers currently hinder this transition:

- The bioinformatics bottleneck: High-throughput sequencing technologies (like metagenomics) generate immense and complex datasets. Interpreting this raw sequence data to produce a clinically meaningful and actionable report requires specialized computational expertise, sophisticated bioinformatics pipelines, and curated reference databases. Most clinical laboratories currently lack the infrastructure, trained personnel, and standardized analytical workflows to effectively handle this “big data” challenge (Lloyd-Price et al., 2016).
- Lack of standardization and validation: There is a critical lack of standardization across the entire microbiota analysis workflow—from sample collection methods and storage conditions to DNA extraction protocols, sequencing platforms, and data analysis pipelines. These variations at different stages can significantly alter the results, making it difficult to compare data between different laboratories or studies and hindering the establishment of robust reference ranges or diagnostic thresholds. Establishing validated, standardized, and quality-controlled protocols is essential for regulatory approval and widespread clinical adoption. Diagnostic tests mentioned in some contexts, like organic acid tests, comprehensive digestive stool analysis (CDSA), or hydrogen breath tests, though provide some insights, are generally less comprehensive than metagenomic approaches for a full microbiome picture (Lloyd-Price et al., 2016) (Proctor et al., 2019).
- Clinical actionability, cost, and reimbursement: The current cost of comprehensive metagenomic sequencing, while decreasing, remains relatively high for routine clinical use. For widespread adoption, the test must not only become more affordable but, more importantly, must provide clinically actionable information. A physician needs a report that goes beyond a simple description of “dysbiosis”; it must offer insights that can guide specific, evidence-based treatment decisions or prognostic assessments. Proving that this information leads to improved patient outcomes and is cost-effective will be the ultimate test of its clinical utility and will be necessary for securing reimbursement from healthcare payers (Lloyd-Price et al., 2016).

For microbiota profiling to become clinically mainstream, it must evolve from simply characterizing dysbiosis to providing predictive, actionable insights that demonstrably improve patient outcomes in a cost-effective manner. This requires the development and validation of robust microbiome-based biomarkers linked to disease risk, diagnosis, prognosis, or prediction of treatment response. Such development, in turn, depends on large, well-controlled clinical studies.

### 7.4 Causality, translational challenges, and precision multi-omics

Despite the rapid advances in microbiome research, a well-established causal links between specific taxa of microorganisms, their metabolites, and defined immune phenotypes have not been determined and it remains a major obstacle in microbiome research (Walter et al., 2020). Majority of the human studies are mainly cross-sectional case–control designs that connects the term “dysbiosis” with disease states, however, it cannot reliably differentiate whether microbiome alterations induce pathology or are secondary effects, nor can they distinguish microbial effects from interfering effects such as medications, diet, or host genetics (Vujkovic-Cvijin et al., 2020). Henceforth, eexperimental studies often use simplified model systems, such as gnotobiotic mice colonized with defined microbial communities or FMT into germ-free or antibiotic-treated animals. These serve as valuable models for understanding the mechanisms involved in host–microbiome interactions under regulated conditions. Nevertheless, they lack the ability to capture completely the complex interactions between human microbiota, immune system, and its environmental exposures. As a result, findings from these models may not always accurately predict human biology or immune development (Fritz et al., 2013).

To overcome these challenges, future research should focus on including long-term human studies, that would clearly monitor changes in microbiota and immune system. Clinical interventions that involve probiotics, diet or defined microbial consortia along with standardized methods for sample collection, processing, sequencing, and data analysis are required to ensure consistent and reproducible findings across different studies (Mirzayi et al., 2021).

Translating microbiota–immune research into clinical applications remains a challenge. Clinical trials involving FMT, probiotics, and live biotherapeutic products have displayed variable results due to variations in microbiome composition, treatment formulations, and patient- specific variables such as immune status, and underlying diseases. These can also influence safety and efficacy of the treatment. Hence, microbiome-based therapeutics requires standardized manufacturing protocols, strict quality control, and appropriate regulatory measures to ensure their safety and effectiveness. Also, microbiome-based biomarkers need to be validated clinically before they can be used in practice for routine applications (McBurney et al., 2019).

Currently emerging multi-omics technologies has improved our understanding of microbiome immune system interactions. Microbial communities and their functional gene expression can be identified using metagenomics and metatranscriptomics analysis, whereas, metabolomics and lipidomics depicts the interactions of the molecules produced by microbes that influence host immunity (Heintz-Buschart and Wilmes, 2018; Lloyd-Price et al., 2019). Integrating these data with host genomics, proteomics, and transcriptomics information along with immunophenotyping provides a more complete understanding of host–microbiome interactions. Machine learning and Artificial intelligence platforms can integrate these complex datasets to identify microbial markers that can predict disease risk or treatment response and to discover new therapeutic targets. Together these approaches can help advance microbiome research by developing personalized, microbiome-based therapies (Topçuoğlu et al., 2020).

### 7.5 Summary of current understanding and translational potential

The microbiota acts as a master regulator of host immunity, engaging in a continuous dialogue through a diverse array of mechanisms. These include the education of the immune system within specialized structures like the GALT, the sensing of microbial MAMPs by host PRRs like TLRs and NLRs, and the active modulation of these signaling pathways by commensal bacteria to promote tolerance. A cornerstone of this communication is the production of microbial metabolites, such as SCFAs, secondary bile acids, and tryptophan catabolites, which act as potent signaling molecules that can program innate and adaptive immune cell functions, often through epigenetic modifications that confer lasting changes. The microbiota meticulously orchestrates the balance between immune tolerance and protective inflammation, influencing the differentiation of T cell subsets like Tregs and Th17 cells, driving mucosal IgA production, and contributing to the formation and maintenance of immunological memory (Belkaid and Harrison, 2017).

Disruptions in this delicate host-microbiota relationship, termed dysbiosis, are implicated in a wide spectrum of conditions, ranging from gastrointestinal disorders like IBD and systemic diseases affecting the lungs (via the gut-lung axis) and the brain (via the gut-brain-immune axis), and even localized infections and potentially cancers in the urogenital tract. Pathogens often exploit or exacerbate dysbiosis to their advantage, creating inflammatory niches that favor their growth over commensals (Belkaid and Harrison, 2017).

This deepening mechanistic understanding is fueling an exciting wave of translational research aimed at harnessing the microbiota for therapeutic benefit. Strategies are rapidly evolving from empirical approaches to more precise interventions. These include next-generation probiotics and engineered LBPs designed to deliver specific immunomodulatory molecules or perform targeted functions; prebiotics to nourish beneficial resident microbes; synbiotics combining these approaches; and postbiotics that deliver the beneficial microbial products directly. Bacteriophage therapy offers a highly specific means to combat MDR pathogens, while FMT and its more refined successors—defined microbial consortia like SER-109 and VE303—aim to restore entire functional ecosystems (Kortright et al., 2019; Feuerstadt et al., 2022; Liu et al., 2023). Furthermore, microbiota-targeted approaches are being explored as adjuncts to improve the efficacy and reduce the side effects of antibiotics and vaccines (Belkaid and Harrison, 2017). The field is rapidly progressing from merely describing the phenomena of microbiota-immune interactions to mechanistically dissecting them and, most importantly, therapeutically manipulating them with increasing precision and sophistication. This trajectory promises to transform medicine, offering novel preventive and therapeutic avenues for a vast array of human diseases that currently lack fully effective treatments.

The journey to fully realize the therapeutic potential of the microbiota is ongoing and requires sustained effort. Key challenges remain, including navigating the profound interindividual variability of human microbiota, which necessitates a shift towards personalized therapeutic strategies. Stringent regulatory, safety, and manufacturing standards must be established for novel LBPs and other microbiota-based products. For personalization to be effective, microbiome profiling techniques need to be standardized, validated, and integrated into routine clinical practice in a way that provides actionable and cost-effective insights. Above all, rigorous, well-designed longitudinal human studies and large-scale RCTs are paramount to establish causality, confirm efficacy, and ensure the safety of these emerging therapies (Proctor et al., 2019).

The future of medicine will undoubtedly involve a more holistic, systems-level understanding of human health, where the microbiota is recognized not as a separate entity but as an integral component of the host organism. Integrating microbiota-based diagnostic and therapeutic approaches into mainstream medical care will require continued cutting-edge research, fostering interdisciplinary collaborations between microbiologists, immunologists, clinicians, bioinformaticians, engineers, and regulatory scientists. By addressing the current challenges and embracing this integrative paradigm, we can unlock the full potential of our microbial partners to prevent disease, enhance existing treatments, and ultimately improve human health on a global scale. The era of microbiome medicine is dawning, promising a new frontier in our quest for health and well-being.

Looking forward, the collective integration of AI-assisted data integration, longitudinal human cohorts, and multi-omics profiling, will be critical to move beyond associative “dysbiosis” signatures toward causal, clinically actionable microbiome-based diagnostics and therapeutics that can be embedded within precision medicine frameworks.

### 7.6 Outstanding questions and future directions

Although mechanistic progress has been achieved in the area of microbiome modulated therapies, several questions remain unanswered and are expected to define the next phase of microbiota research.

- Strain-level specificity: Majority of the immunomodulatory effects discussed here are associated at the genus or species level, however, closely related strains can produce considerable variation, even displaying opposing effects. Systematic starin resolved functional studies are required to assess the immune effects.
- Rational consortium design: Although defined multi-strain consortia (e.g., SER-109, VE303) have displayed promising therapeutic outcomes, it is essential to assess whether they outperform single-strain LBPs in a consistent manner, as the criteria involved for complementary strain selection remains largely empirical.
- Microbiome-based biomarkers: Despite recent advances, a reproducible and validated, microbial or metabolite markers that predict disease risk or treatment response are still lacking for most diseases.
- Longitudinal human studies: Majority of the research on microbiome is based on cross sectional studies that can identify associations between the microbiome and disease and provide data at a single time point. However, they cannot differentiate between microbial changes as a cause or consequence of disease. Integrating multi-omics approaches such as metagenomics and metabolomics on large data are required to track these changes over time. These studies will improve our understanding of disease mechanisms and aids in the development of reliable biomarkers and microbiome-based therapies.
- AI-assisted microbiome analysis: The machine learning models are highly dependent on the availability of standardized training models and needs thorough validation using independent external cohorts before it is being deployed for clinical purposes.
- Regulatory harmonization: Despite the regulatory guidelines developed by agencies such as the FDA and EMA for microbiome-based therapeutics, a lack of unified standards for postbiotics, live biotherapeutics, and defined microbial consortia remains, leading to uncertainty in product development and clinical implementation. A unified frameworks for these microbiome derived products need to be implemented by these regulatory agencies for effective translational development.

### 7.7 Conclusion: harnessing the microbiome for future health

The exploration of the host-microbiota interface has unveiled a universe of intricate interactions that are fundamental to health and disease. It is now unequivocally clear that the vast communities of microorganisms inhabiting our bodies are not passive passengers but active participants in shaping our physiology, particularly the development, function, and regulation of our immune system.

## Glossary

Treg: Regulatory T cells
Th17: T helper 17 cells
IgA: Immunoglobulin A
DCs: Dendritic cells
IL-10: Interleukin- 10
TGF-β: Transforming growth factor-beta
MAMPs: Microbe-Associated Molecular Patterns
PAMPs: Pathogen-Associated Molecular Patterns
AMPs: Antimicrobial peptides
sIgA: Secretory IgA
GALT: Gut-Associated Lymphoid Tissues
SCFAs: Short Chain Fatty Acids
SFB: Segmented Filamentous Bacteria
LPS: Lipopolysaccharides
TLR: Toll-like receptor
NLR: NOD-like receptor
HDAC: Histone Deacetylases
ROS: Reactive Oxygen Species
TNF-α: Tumor Necrosis Factor – alpha
GPR43: G protein-coupled receptor
ARG1: arginase-1
PSA: Polysaccharide A
TGR5-: Takeda G protein-coupled receptor 5
DAT: Desaminotyrosine
IFN-I: Type-I Interferon
STING pathway: Stimulator of Interferon Genes
IRAK-M: Interleukin-1 Receptor-Associated Kinase M
IBD: Inflammatory Bowel Disease
NK: Natural Killer cells
IECs: Intestinal Epithelial Cells
AhR: Aryl Hydrocarbon Receptor
HIF: Hypoxia-Inducible Factor
DCA: Deoxycholic Acid
LCA: Lithocholic Acid
FXR: Farnesoid X Receptor
IDO1: Indoleamine 2, 3-dioxygenase
TDO: Tryptophan 2, 3-dioxygenase
SAM: S-adenosylmethionine
ARNT: AhR Nuclear Translocator
XREs: Xenobiotic Response Elements
HATs: Histone Acetyltransferases
MALT: Mucosal-Associated Lymphoid Tissue
CSR: Class Switch Recombination
Tfh: T Follicular Helper cells
BAFF/APRIL: B-cell Activating Factor/A Proliferation Inducing Ligand
TSLP: Thymic stromal lymphopoietin
ILFs: Isolated Lymphoid Follicles
PPs: Peyer’s Patches
MLNs: Mesenteric Lymph Nodes
FAE: Follicle-Associated Epithelium
SED: Subepithelial Dome
CDI: *Clostridioides difficile* Infection
SPIs: Salmonella Pathogenicity Islands
CPE: Carbapenemase-producing *Enterobacterales*
LAP: Listeria Adhesion Protein
AdhE: Acetaldehyde Alcohol Dehydrogenase
FMT: Fecal Microbiota Transplantation
GVHD: Graft Versus Host Disease
HGT: Horizontal Gene Transfer
CDSA: Comprehensive Digestive Stool Analysis
MDR: Multi Drug Resistant
